# Differential Metabolic Responses of Lettuce Grown in Soil, Substrate and Hydroponic Cultivation Systems under NH_4_^+^/NO_3_^−^ Application

**DOI:** 10.3390/metabo12050444

**Published:** 2022-05-16

**Authors:** Muhammad Khalid Hameed, Wajid Umar, Ali Razzaq, Tariq Aziz, Muhammad Aamer Maqsood, Shiwei Wei, Qingliang Niu, Danfeng Huang, Liying Chang

**Affiliations:** 1 School of Agriculture and Biology, Shanghai Jiao Tong University, Shanghai 200240, China; khalid_khalid45@yahoo.com (M.K.H.); qlniu@sjtu.edu.cn (Q.N.); hdf@sjtu.edu.cn (D.H.); 2 Institute of Environmental Science, Hungarian University of Agriculture and Life Sciences, 2100 Gödöllő, Hungary; wajidumar30@gmail.com; 3 Centre of Agricultural Biochemistry and Biotechnology (CABB), University of Agriculture, Faisalabad 38000, Pakistan; ali.razzaq254@gmail.com; 4 Institute of Soil and Environmental Sciences, University of Agriculture, Faisalabad 38000, Pakistan; draziz@uaf.edu.pk (T.A.); mohamgill@uaf.edu.pk (M.A.M.); 5 Shanghai Agrobiological Gene Center, Shanghai 201106, China; wsw@sagc.org.cn

**Keywords:** ammonium, lettuce, metabolites, nitrogen, nitrate

## Abstract

Nitrogen (N) is an essential element for plant growth and development. The application of a balanced and optimal amount of N is required for sustainable plant yield. For this, different N sources and forms are used, that including ammonium (NH_4_^+^) and nitrate (NO_3_^−^). These are the main sources for N uptake by plants where NH_4_^+^/NO_3_^−^ ratios have a significant effect on the biomass, quality and metabolites composition of lettuce grown in soil, substrate and hydroponic cultivation systems. A limited supply of N resulted in the reduction in the biomass, quality and overall yield of lettuce. Additionally, different types of metabolites were produced with varying concentrations of N sources and can be used as metabolic markers to improve the N use efficiency. To investigate the differential metabolic activity, we planted lettuce with different NH_4_^+^/NO_3_^−^ ratios (100:0, 75:25, 50:50, 25:75 and 0:100%) and a control (no additional N applied) in soil, substrate and hydroponic cultivation systems. The results revealed that the 25% NH_4_^+/^75% NO_3_^−^ ratio increased the relative chlorophyll contents as well as the biomass of lettuce in all cultivation systems. However, lettuce grown in the hydroponic cultivation system showed the best results. The concentration of essential amino acids including alanine, valine, leucine, lysine, proline and serine increased in soil and hydroponically grown lettuce treated with the 25% NH_4_^+/^75% NO_3_^−^ ratio. The taste and quality-related compounds in lettuce showed maximum relative abundance with the 25% NH_4_^+/^75% NO_3_^−^ ratio, except ascorbate (grown in soil) and lactupicrin (grown in substrate), which showed maximum relative abundance in the 50% NH_4_^+/^50% NO_3_^−^ ratio and control treatments, respectively. Moreover, 1-O-caffeoylglucose, 1,3-dicaffeoylquinic acid, aesculetin and quercetin-3-galactoside were increased by the application of the 100% NH_4_^+/^0% NO_3_^−^ ratio in soil-grown lettuce. The 25% NH_4_^+/^75% NO_3_^−^ ratio was more suitable in the hydroponic cultivation system to obtain increased lettuce biomass. The metabolic profiling of lettuce showed different behaviors when applying different NH_4_^+^/NO_3_^−^ ratios. Therefore, the majority of the parameters were largely influenced by the 25% NH_4_^+/^75% NO_3_^−^ ratio, which resulted in the hyper-accumulation of health-promoting compounds in lettuce. In conclusion, the optimal N applications improve the quality of lettuce grown in soil, substrate and hydroponic cultivation systems which ultimately boost the nutritional value of lettuce.

## 1. Introduction

Nitrogen (N) is considered an essential element for plant growth and development. It is the major constituent of amino acids, which are the building blocks of proteins and is involved in the catalyzation of chemical responses and the transportation of electrons. It is also present in chlorophyll (enabling the process of photosynthesis) in the plant body. The amount of N directly affects plant growth and yield, which are often affected by N deficiency [1]. In most soil, N is a limiting nutrient required for the maximum production of crops [2]. To overcome N deficiency, an excessive amount of N is applied externally as fertilizer. However, the addition of excess amounts of N to plants for yield improvements is not always beneficial. Excessive N application reduces the N use efficiency in plants and causes detrimental effects on plants and the environment e.g., eutrophication, acid rain and the greenhouse gas effect [3,4,5]. Thus, the optimal use of N fertilizers using different strategies is of prime interest to reduce the negative effects on plants and the environment [6].

Preference for N uptake depends upon the plant species, the plant development stage and environmental growing conditions [7]. N is mainly available in the form of ammonium (NH_4_^+^) and nitrate (NO_3_^−^) to the plants. Functionally, NH_4_^+^ and NO_3_^−^ are involved in regulating plant physiology, growth and development processes, including plant biomass, root and shoot length, seed germination, growth of roots and leaves, the structure of roots, flowering time, senescence and the yields of plants [8,9,10]. In plants, lower NO_3_^−^ concentrations and higher NH_4_^+^ concentrations promote early flowering while higher NO_3_^−^ concentrations and lower NH_4_^+^ concentrations delay flowering time [11,12]. Therefore, appropriate N management practices, such as N application methods and application rates to the plants in agroecosystems are considered an important component of achieving the best results and minimizing environmental risks [13].

Lettuce is a leafy vegetable grown all over the world due to its nutritional value. Generally, lettuce is grown in different cultivation systems, such as hydroponic, soil and substrate systems, to fulfill the daily requirements of humans. It contains natural health-promoting compounds that play an important role in preventing many chronic illnesses [14,15] and inhibiting tumorigenesis and metastasis [16]. Lettuce also contains anti-fungal, anti-bacterial and anti-inflammatory capabilities that helps to delay the aging process in humans [17]. Different factors influence health-promoting compounds in plants, such as water application, agronomic practices, the growing medium, fertilization, environmental factors and harvesting time [18,19,20,21].

In a hydroponic cultivation system, plant growth and development occur in a controlled environment by regulating different factors, such as the growing medium, light duration and plant nutrition [22,23]. Additionally, the initial costs to establish the hydroponic cultivation system to grow and obtain the desired economic yield are much higher compared to soil and substrate cultivation systems [24,25]. Nutrients are directly taken up by plants and stimulate the composition of different phytochemicals, such as ascorbic acid, phenolic compounds and flavonoids in plants [10,26]. The plant takes up nutrients through its roots with little effort and makes full use of its energy to grow tissues as compared to the soil system [27,28]. In contrast, plants growing in soil and substrate cultivation systems benefit from soil nutrient mineralization and microbial activity [29]

Metabolic changes are used as a quality evaluation for agricultural products and are also used to identify different metabolites under different growing conditions [30,31,32]. Approximately 200,000 metabolites with defined structures are produced by different species of plants in different ecosystems [33]. Metabolites are categorized into two groups: primary and secondary metabolites. High-throughput metabolomics data acquisition through gas chromatography-mass spectrometry (GC-MS) and liquid chromatography-mass spectrometry (LC-MS) provides highly sensitive results to identify unique metabolites [34]. Untargeted metabolomic profiling through GC-MS and LC-MS is considered a promising analytical method to determine the taste, quality and functional characteristics of plants and agricultural products [35]. In addition, the combination of different mass spectrometry-based techniques explores the vast range of different metabolites in plants as compared to a single mass spectrometry technique [36]. These techniques are also used to identify qualitative and quantitative variations in the metabolic profiling of plants. Taken together, variations in the metabolic profiles of lettuce grown in different regimes of N nutrition in different cultivation systems is very important for improving the yield, quality and nutritional values of lettuce.

A few studies on the composition of lettuce metabolites in hydroponic systems have been reported [37,38,39]. It has not been fully explored how different NH_4_^+^/NO_3_^−^ ratios affect the sugar compounds, amino acid contents and polyphenolic compounds in lettuce and how these metabolic changes influence nutritional values in lettuce. In this study, we aimed to explore the metabolic response of lettuce by applying different NH_4_^+^/NO_3_^−^ ratios in soil, substrate and hydroponic cultivation systems.

## 2. Results

### 2.1. Physiological and Growth Parameters of Lettuce

**Figure 1:**
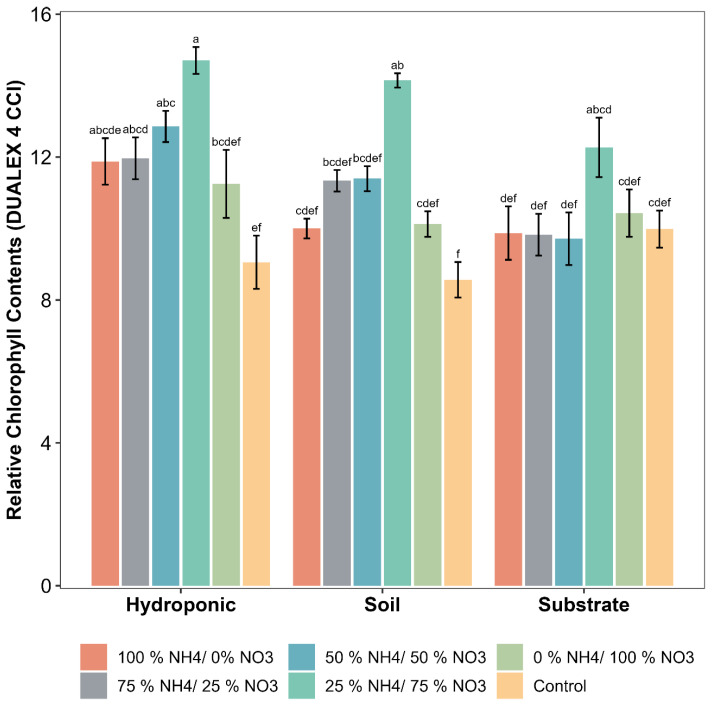
Effect of various applied NH_4_^+^/NO_3_^−^ ratios on the relative chlorophyll contents of lettuce grown in soil, substrate and hydroponic cultivation systems. Note- The means (*n* = 4) ± standard errors are shown in the data. Different letters (a–f) show significant difference (*p* = 0.01) among applied NH_4_^+^/NO_3_^−^ ratios (100:0, 75:25, 50:50, 25:75 and 0:100%) and control (no additional N applied) in lettuce grown in soil, substrate and hydroponic cultivation systems.

The relative chlorophyll contents of lettuce were significantly influenced by the applied NH_4_^+^/NO_3_^−^ ratios as compared to the control treatment (Figure 1). The maximum relative chlorophyll contents were observed with 25% NH_4_^+/^75% NO_3_^−^ ratio in all of the cultivation systems used in this experiment. The increase in the relative chlorophyll contents as compared to the control in soil, substrate and hydroponic cultivation systems was 64.43, 22.94 and 63.78% respectively. In the case of the hydroponic cultivation system, the 25% NH_4_^+/^75% NO_3_^−^ and 50% NH_4_^+/^50% NO_3_^−^ ratios showed a significant difference as compared to the control. Other applied NH_4_^+^/NO_3_^−^ ratios did not show a significant difference as compared to the control. Among cultivation systems, hydroponically grown lettuce showed the maximum increase followed by soil, in the relative chlorophyll contents, irrespective of applied treatment, as compared to substrate cultivation system.

**Figure 2:**
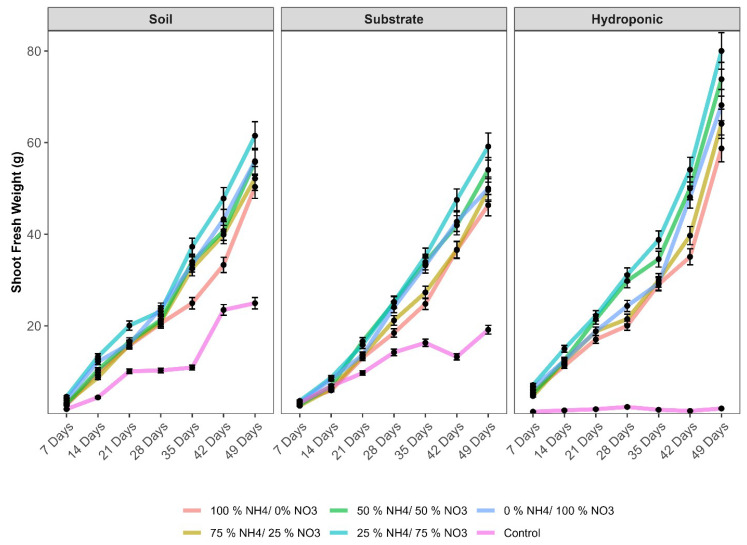
Effect of various applied NH_4_^+^/NO_3_^−^ ratios (100:0, 75:25, 50:50, 25:75 and 0:100%) and control (no additional N applied) in different intervals of time on shoot fresh biomass of lettuce grown in soil, substrate and hydroponic cultivation systems. Note-The means (*n* = 4) ± standard errors are shown in the data.

The shoot fresh biomass increased significantly as the plant reached maturity (Figure 2). Maximum shoot fresh biomasses of 80.0, 61.5 and 45.9 g were recorded on the 49th day with the 25% NH_4_^+/^75% NO_3_^−^ ratio in hydroponic, soil, and substrate, respectively. Among the cultivation systems, the maximum shoot fresh biomass was observed in the hydroponic system as compared to the others.

### 2.2. Taste and Quality-Related Compounds

**Figure 3:**
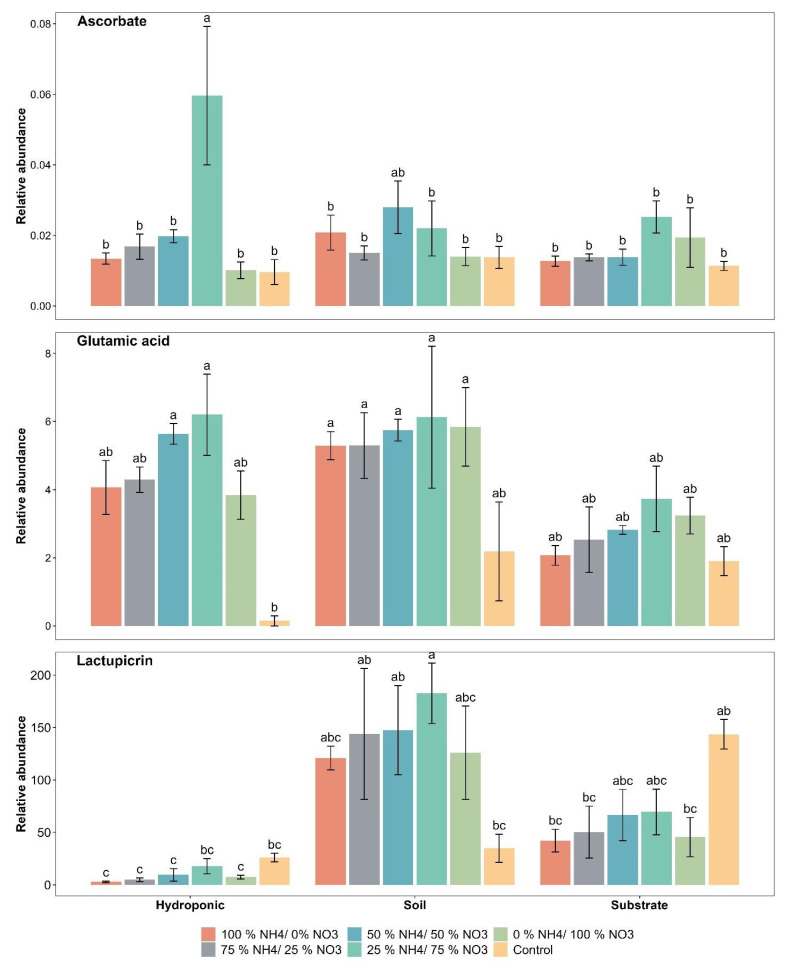
Effect of various applied NH_4_^+^/NO_3_^−^ ratios (100:0, 75:25, 50:50, 25:75 and 0:100%) and control (no additional N applied) on taste and quality-related compounds in lettuce grown in soil, substrate and hydroponic cultivation systems. Note- The means (*n* = 4) ± standard errors are shown in the data. Different letters (a–c) show significant difference (*p* = 0.01) among applied NH_4_^+^/NO_3_^−^ ratios (100:0, 75:25, 50:50, 25:75 and 0:100%) and control in lettuce grown in soil, substrate and hydroponic cultivation systems.

The relative abundance of ascorbate was found to range from 0.009 to 0.059 for the applied treatments in lettuce grown in all three cultivation systems. The relative abundance of ascorbate (0.059) was significantly improved by 25% NH_4_^+/^75% NO_3_^−^ in hydroponically grown lettuce. However, the 50% NH_4_^+/^50% NO_3_^−^ ratio significantly increased the relative abundance of ascorbate (0.027) in soil-grown lettuce as compared to the control (Figure 3). Among cultivation systems, hydroponically grown plants showed the maximum increase in the relative abundance of ascorbate but did not show a significant difference in the relative abundance of ascorbate, irrespective of the applied treatment, as compared to soil and substrate systems.

The relative abundance of glutamic acid was significantly increased by the applied NH_4_^+^/NO_3_^−^ ratios in hydroponically and soil-grown lettuce but did not show a significant difference in the substrate system (Figure 3). The maximum relative abundance of glutamic acid was observed with the 25% NH_4_^+/^75% NO_3_^−^ ratio in lettuce grown in all three cultivation systems. The increase in the relative abundance of glutamic acid with the 25% NH_4_^+/^75% NO_3_^−^ ratio was about 64.27, 49.01 and 97.61% as compared to the control in soil-, substrate- and hydroponically grown lettuce.

The relative abundance of lactupicrin was found to range from 2.85 to 182.68 with the applied treatments in lettuce grown in all three cultivation systems. The relative abundance of lactupicrin (182.68) was significantly improved by 25% NH_4_^+/^75% NO_3_^−^ in the soil system as compared to the control (Figure 3). However, a significant decrease in the relative abundance of lactupicrin was observed with the applied NH_4_^+^/NO_3_^−^ ratios as compared to the control in the substrate- and hydroponically grown lettuce. In substrate and hydroponic systems, 51% and 31% decreases in the relative abundance of lactupicrin with the 25% NH_4_^+/^75% NO_3_^−^ ratio was observed as compared to the control, respectively. The lettuce grown in the three cultivation systems showed a significant difference in the relative abundance of lactupicrin.

### 2.3. Sugar Compounds

**Figure 4:**
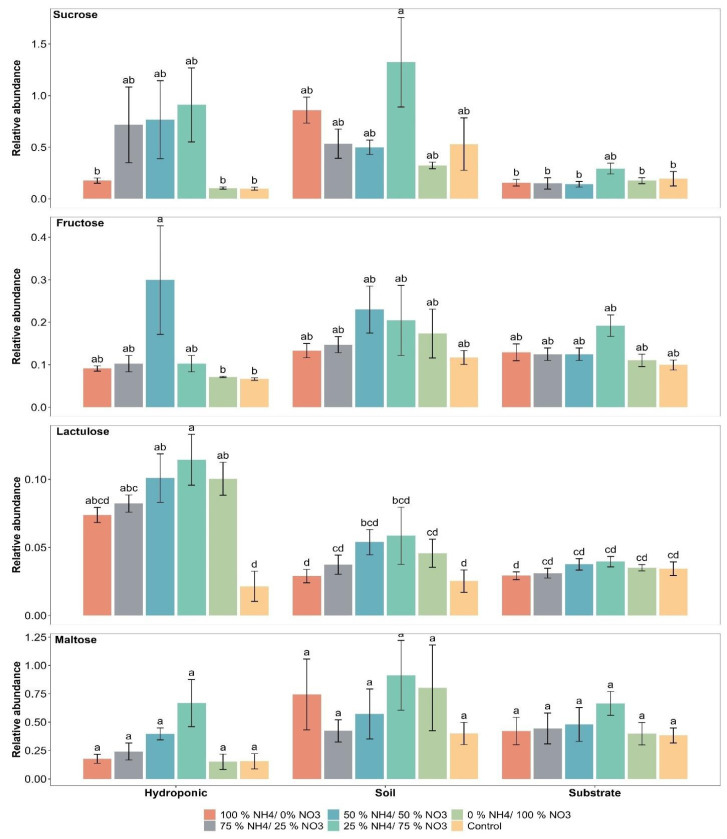
Effect of various applied NH_4_^+^/NO_3_^−^ ratios (100:0, 75:25, 50:50, 25:75 and 0:100%) and control (no additional N applied) on sugar compounds in lettuce grown in soil, substrate and hydroponic cultivation systems. Note- The means (*n* = 4) ± standard errors are shown in the data. Different letters (a–d) show significant difference (*p* = 0.01) among applied NH_4_^+^/NO_3_^−^ ratios (100:0, 75:25, 50:50, 25:75 and 0:100%) and control in lettuce grown in soil, substrate and hydroponic cultivation systems.

The relative abundance of sucrose was found to range from 0.09 to 1.32 with the applied treatments in lettuce grown in all three cultivation systems. A significant difference in the relative abundance of sucrose in lettuce was found with the 25% NH_4_^+/^75% NO_3_^−^ ratio as compared to the control in soil, substrate and hydroponic systems (Figure 4). The maximum relative abundance of sucrose was 1.32, 0.29 and 0.90 with the 25% NH_4_^+/^75% NO_3_^−^ ratio in soil, substrate and hydroponically grown lettuce, respectively. However, the other applied NH_4_^+^/NO_3_^−^ ratios did not show a significant difference in the relative abundance of sucrose as compared to control in soil and substrate systems. In addition, NH_4_^+^/NO_3_^−^ ratios of 25/75, 50/50 and 75/25% showed a significant difference in the relative abundance of sucrose as compared to the control in the hydroponic system. Lettuce grown in soil showed a significant difference, followed by hydroponically grown lettuce, in the relative abundance of sucrose, irrespective of applied treatment, as compared to the substrate system.

The relative abundance of fructose was found to range from 0.06 to 0.29 with the applied treatments in lettuce grown in all three cultivation systems. The maximum increases in the relative abundance of fructose of 0.29 and 0.22 were recorded with the 50% NH_4_^+/^50% NO_3_^−^ ratio in hydroponic and soil cultivation systems, respectively (Figure 4). However, the maximum increase in the relative abundance of fructose (0.19) was observed with the 25% NH_4_^+/^75% NO_3_^−^ ratio in the substrate cultivation system. The applied NH_4_^+^/NO_3_^−^ ratios did not show a significant difference in the relative abundance of fructose as compared to the control in soil and substrate cultivation systems. A significant difference was observed with the 25% NH_4_^+/^75% NO_3_^−^ ratio as compared to the control in the hydroponic cultivation system. The lettuce grown in the three cultivation systems did not show a significant difference in the relative abundance of fructose when compared to each other.

A significant difference in the relative abundance of lactulose was observed with the 25% NH_4_^+/^75% NO_3_^−^ ratio in soil- (132%) and hydroponically grown lettuce (442.85%) as compared to the control (Figure 4). Furthermore, the applied NH_4_^+^/NO_3_^−^ ratios showed a significant difference in the relative abundance of lactulose in lettuce as compared to the control in hydroponic system. In addition, the applied NH_4_^+^/NO_3_^−^ ratios did not show a significant difference in the relative abundance of lactulose in lettuce, except for the 100% NH_4_^+/^0% NO_3_^−^ ratio, as compared to the control in the substrate system. The lettuce grown in the hydroponic system showed a significant difference in the relative abundance of lactulose as compared to the others.

The relative abundance of maltose was found to range from 0.15 to 0.91 with the applied treatments in lettuce grown in all three cultivation systems. The maximum relative abundance of maltose (0.91) was observed with the 25% NH_4_^+/^75% NO_3_^−^ ratio in the soil-grown lettuce (Figure 4). The relative abundance of maltose was about 0.66 and 0.66 in the substrate- and hydroponically grown lettuce, respectively. The applied NH_4_^+^/NO_3_^−^ ratios did not show a significant difference in the relative abundance of maltose in soil-, substrate- and hydroponically grown lettuce. Lettuce grown in soil showed a significant difference, followed by lettuce grown in substrate cultivation system, in the relative abundance of maltose as compared to the hydroponic system.

### 2.4. Amino Acid Contents

**Figure 5:**
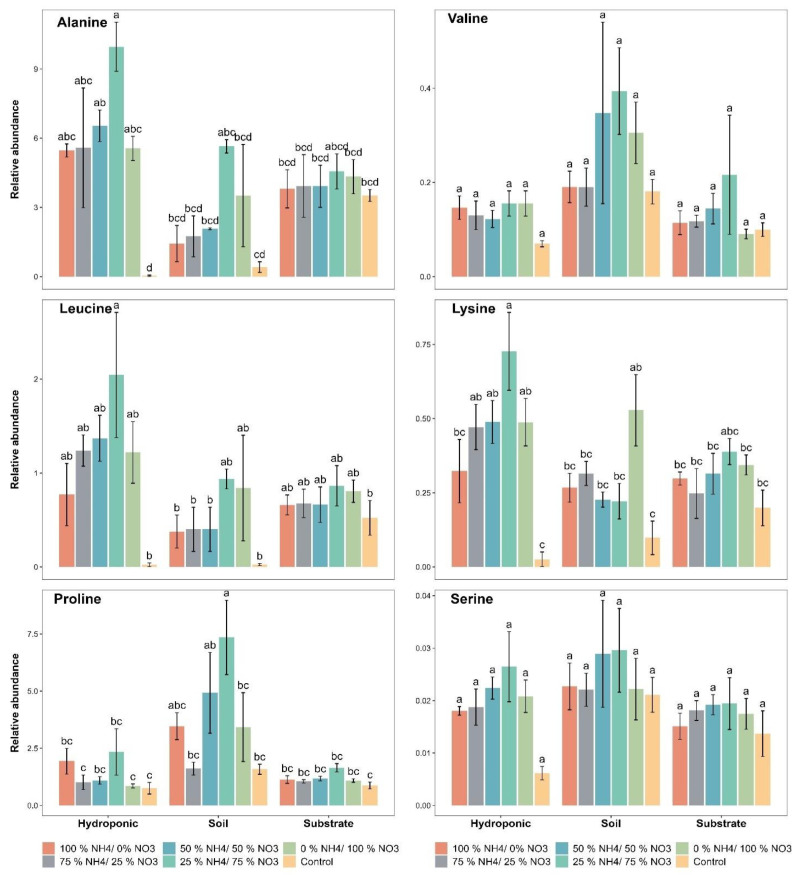
Effect of various applied NH_4_^+^/NO_3_^−^ ratios (100:0, 75:25, 50:50, 25:75 and 0:100%) and control (no additional N applied) on different amino acid contents of lettuce grown in soil, substrate and hydroponic cultivation systems. Note- The means (*n* = 4) ± standard errors are shown in the data. Different letters (a–d) show significant difference (*p* = 0.02) among applied NH_4_^+^/NO_3_^−^ ratios (100:0, 75:25, 50:50, 25:75 and 0:100%) and control in lettuce grown in soil, substrate and hydroponic cultivation systems.

Amino acid contents were influenced by the applied NH_4_^+^/NO_3_^−^ ratios in all cultivation systems. The relative abundance of alanine was found to range from 0.03 to 9.96 with the applied treatments in lettuce grown in all three cultivation systems. Alanine had a maximum relative abundance of 5.64, 4.55 and 9.96 with the 25% NH_4_^+/^75% NO_3_^−^ ratio in soil-, substrate- and hydroponically grown lettuce, respectively (Figure 5). A significant difference in the relative abundance of alanine was found with the applied NH_4_^+^/NO_3_^−^ ratios as compared to the control in lettuce grown in the three cultivation systems. Furthermore, lettuce grown in the three cultivation systems showed a significant difference in the relative abundance of alanine, irrespective of the applied treatment, when compared to each other.

The relative abundance of valine was found to range from 0.09 to 0.19 with the applied treatments in lettuce grown in all three cultivation systems. The maximum relative abundance of valine (0.39) was found in soil-grown lettuce with the 25% NH_4_^+/^75% NO_3_^−^ ratio (Figure 5). Overall, the 25% NH_4_^+/^75% NO_3_^−^ ratio performed well among the applied NH_4_^+^/NO_3_^−^ ratios for the relative abundance of valine in soil-, substrate- and hydroponically grown lettuce. None of the applied NH_4_^+^/NO_3_^−^ ratios showed a significant difference in the relative abundance of valine as compared to the control in all three systems. The soil-grown lettuce showed a significant difference in the relative abundance of valine as compared to substrate and hydroponic systems.

A significant difference in the relative abundance of leucine was observed with the 25% NH_4_^+/^75% NO_3_^−^ ratio as compared to the control in soil-, substrate- and hydroponically grown lettuce (Figure 5). However, the other applied NH_4_^+^/NO_3_^−^ ratios did not show a significant difference in the relative abundance of leucine when compared to each other in hydroponic and substrate systems. Hydroponically grown lettuce showed a significant difference in the relative abundance of leucine as compared to soil and substrate systems.

The relative abundance of lysine was found to be significantly higher (0.72 and 0.38) with the 25% NH_4_^+/^75% NO_3_^−^ ratio in hydroponically and substrate-grown lettuce, respectively (Figure 5). In addition, a significant difference in the relative abundance of lysine in lettuce was observed with the 25% NH_4_^+/^75% NO_3_^−^ ratio as compared to the control in hydroponic and substrate systems. Additionally, the 0% NH_4_^+/^100% NO_3_^−^ ratio significantly increased the relative abundance of lysine as compared to the control in soil-grown lettuce. Hydroponically grown lettuce showed a significant difference in the relative abundance of lysine as compared to soil and substrate systems.

A higher relative abundance of proline was observed (364%, 88.5% and 212%) with the 25% NH_4_^+/^75% NO_3_^−^ ratio as compared to the control in soil-, substrate- and hydroponically grown lettuce, respectively (Figure 5). Statistically, the soil-grown lettuce showed a significant difference in the relative abundance of proline as compared to substrate and hydroponic systems.

The relative abundance of serine was increased by 38%, 41.6% and 321.9% with the 25% NH_4_^+/^75% NO_3_^−^ ratio as compared to the control in soil-, substrate- and hydroponically grown lettuce, respectively (Figure 5). The other applied NH_4_^+^/NO_3_^−^ ratios did not show a significant difference in the relative abundance of serine as compared to the control in lettuce grown in the three cultivation systems. Statistically, lettuce grown in the soil showed a significant difference, followed by lettuce grown in hydroponic system, in the relative abundance of serine as compared to the substrate system.

### 2.5. Polyphenolic Compounds

**Figure 6:**
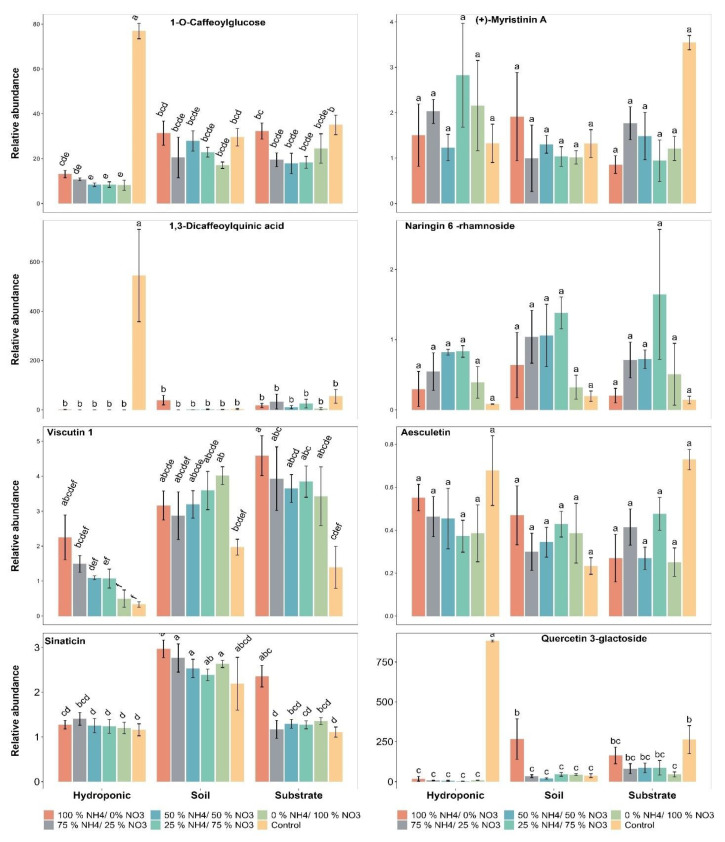
Effect of various applied NH_4_^+^/NO_3_^−^ ratios (100:0, 75:25, 50:50, 25:75 and 0:100%) and control (no additional N applied) on different polyphenolic compounds in lettuce grown in soil, substrate and hydroponic cultivation systems. Note- The means (*n* = 4) ± standard errors are shown in the data. Different letters (a–f) show significant difference (*p* = 0.001) among applied NH_4_^+^/NO_3_^−^ ratios (100:0, 75:25, 50:50, 25:75 and 0:100%) and control in lettuce grown in soil, substrate and hydroponic cultivation systems.

The relative abundance of 1-O-caffeoylglucose (0.06%) was increased with the 100% NH_4_^+/^0% NO_3_^−^ ratio as compared to the control in soil grown lettuce (Figure 6). The applied NH_4_^+^/NO_3_^−^ ratios had less influence on the relative abundance of 1-O-caffeoylglucose as compared to the control in soil-, substrate- and hydroponically grown lettuce. The lettuce grown in the three cultivation systems did not show a significant difference in the relative abundance of 1-O-caffeoylglucose, irrespective of applied treatment, when compared to each other.

The relative abundance of (+)-myristinin A did not show a significant difference with the applied NH_4_^+^/NO_3_^−^ ratios as compared to the control in soil-, substrate- and hydroponically grown lettuce (Figure 6). The lettuce grown in the three cultivation systems did not show a significant difference in the relative abundance of (+)-myristinin A when compared to each other.

A significant difference in the relative abundance of 1,3-dicaffeoylquinic acid was found in the control treatment as compared to the applied NH_4_^+^/NO_3_^−^ ratios in hydroponically grown lettuce (Figure 6). The applied NH_4_^+^/NO_3_^−^ treatments did not show a significant difference in the relative abundance of 1,3-dicaffeoylquinic acid as compared to the control in substrate- and soil-grown lettuce. The lettuce grown in the hydroponic system showed a significant difference in the relative abundance of 1,3-dicaffeoylquinic acid, irrespective of the applied treatment, as compared to soil and substrate systems.

The maximum relative abundance of naringin 6’’-rhamnoside in lettuce was found to be 1.38, 1.64 and 0.83 with the 25% NH_4_^+/^75% NO_3_^−^ ratio in soil-, substrate- and hydroponically grown lettuce, respectively (Figure 6). The applied NH_4_^+^/NO_3_^−^ ratios did not show a significant difference in the relative abundance of naringin 6’’-rhamnoside as compared to the control in lettuce grown in the three cultivation systems. The lettuce grown in the three cultivation systems did not show a significant difference in the relative abundance of naringin 6’’-rhamnoside when compared to each other.

The relative abundance of viscutin 1 was significantly influenced and increased by 22.9% and 57.9% with the 100% NH_4_^+/^0% NO_3_^−^ ratio as compared to the control in the substrate and hydroponically grown lettuce, respectively (Figure 6). The applied 0% NH_4_^+/^100% NO_3_^−^ ratio increased the relative abundance of viscutin 1 by 10.3% as compared to control in soil-grown lettuce. Additionally, the applied NH_4_^+^/NO_3_^−^ ratios increased the relative abundance of viscutin 1 as compared to the control in lettuce grown in the three cultivation systems. The lettuce grown in soil and substrate systems showed a significant difference in the relative abundance of viscutin 1 as compared to the hydroponic system.

The relative abundance of aesculetin did not show a significant difference with the applied NH_4_^+^/NO_3_^−^ ratios as compared to the control in soil-, substrate- and hydroponically grown lettuce (Figure 6). The lettuce grown in the three cultivation systems did not show a significant difference in the relative abundance of aesculetin, irrespective of the applied treatment, when compared to each other.

The applied 100% NH_4_^+/^0% NO_3_^−^ ratio showed a significant difference and increased the relative abundance of sinaticin by 35.77% and 113.63% as compared to the control in soil- and substrate-grown lettuce, respectively (Figure 6). In addition, the applied NH_4_^+^/NO_3_^−^ ratios increased the relative abundance of sinaticin as compared to the control in soil and substrate grown lettuce. Furthermore, the relative abundance of sinaticin was increased by 21.73% with the 75% NH_4_^+/^25% NO_3_^−^ ratio as compared to the control in hydroponically grown lettuce. The lettuce grown in soil showed a significant difference in the relative abundance of sinaticin, irrespective of the applied treatment, as compared to the substrate and hydroponic systems.

The applied 100% NH_4_^+/^0% NO_3_^−^ ratio showed a significant difference and increased the relative abundance of quercetin-3-galactoside by 601.25% as compared to the control in soil-grown lettuce (Figure 6). However, all applied NH_4_^+^/NO_3_^−^ ratios had less of an influence on the relative abundance of quercetin-3-galactoside as compared to the control in hydroponically and substrate-grown lettuce. Furthermore, lettuce grown in the hydroponic system showed a significant difference in the relative abundance of quercetin-3-galactoside, followed by lettuce grown in the substrate system, as compared to the soil system.

### 2.6. Metabolomic Profiling of Lettuce Treated with Different NH_4_^+^/NO_3_^−^ Ratios in Soil, Substrate and Hydroponic Cultivation System

**Figure 7:**
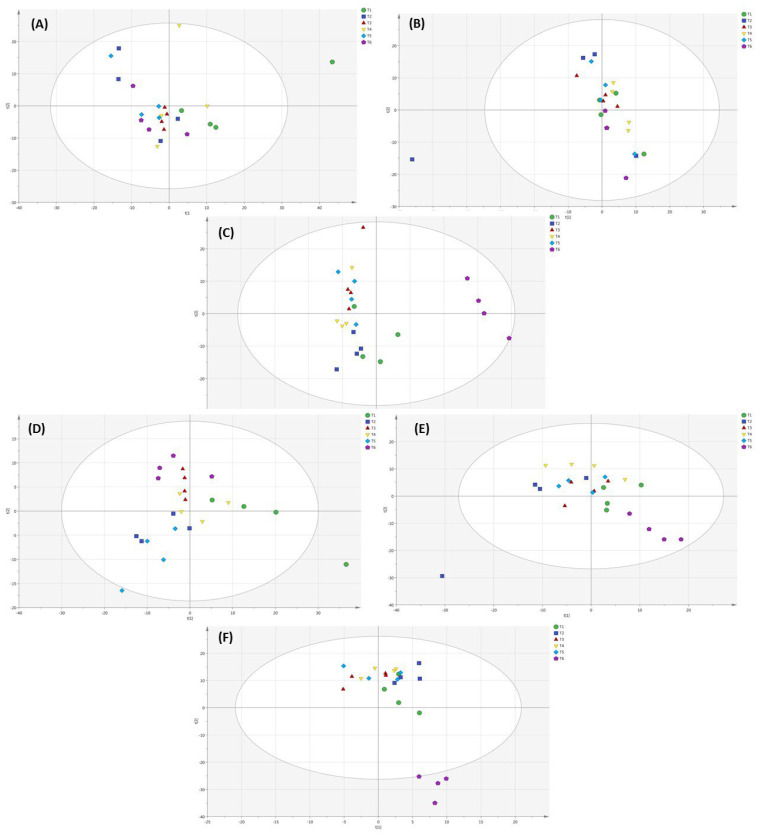
PCA and PLS-DA analysis of the metabolomic profile of lettuce with different applied NH_4_^+^/ NO_3_^−^ ratios (100:0, 75:25, 50:50, 25:75 and 0:100%) and control (no additional N applied) in soil, substrate and hydroponic cultivation systems by GC-MS analysis. Note- (**A**–**C**) represents PCA analysis and (**D**–**F**) represents PLS-DA analysis of metabolomic profiling of lettuce in soil, substrate and hydroponic cultivation systems by GC-MS analysis. Green color represents NH_4_^+^/NO_3_^−^ (100/0%), blue color represents NH_4_^+^/NO_3_^−^ (75/25%), red color represents NH_4_^+^/NO_3_^−^ (50/50%), yellow color represents NH_4_^+^/NO_3_^−^ (25/75%), sky-blue color represents NH_4_^+^/NO_3_^−^ (0/100%) and purple color represents control.

Lettuce metabolites in the soil cultivation system measure by GC-MS showed that different applied NH_4_^+^/NO_3_^−^ ratios affected the metabolomic profile of lettuce (Figure 7A). In PCA, the 100% NH_4_^+/^0% NO_3_^−^ ratio showed a significant difference from other applied NH_4_^+^/NO_3_^−^ treatments and was found in the positive quadrant on the *x*-axis. However, other applied NH_4_^+^/NO_3_^−^ ratios (75:25, 50:50, 25:75, 0:100%) were intermixed with each other and were not clearly separated. This observation suggests that there is little metabolite difference in soil-grown lettuce among applied NH_4_^+^/NO_3_^−^ ratios (100:0, 75:25, 50:50, 25:75 and 0:100%) as treatments. The PCA score plot of substrate-grown lettuce showed that there was less difference in the metabolomic profiles of lettuce among various applied NH_4_^+^/NO_3_^−^ ratios (100:0, 75:25, 50:50, 25:75, 0:100%) and the control (Figure 7B). The 100% NH_4_^+/^0% NO_3_^−^ and 25% NH_4_^+/^75% NO_3_^−^ ratios were found in the positive quadrant on the *x*-axis. Furthermore, the other applied NH_4_^+^/NO_3_^−^ ratios were observed in the negative quadrant on the *x*-axis for substrate-grown lettuce. The PCA analysis of lettuce grown in the hydroponic system is shown (Figure 7C). The metabolomic profile of lettuce with applied NH_4_^+^/NO_3_^−^ ratios (100:0, 75:25, 50:50, 25:75 and 0:100%) appeared in the negative quadrant on the *x*-axis as compared to the control. The 50% NH_4_^+/^50% NO_3_^−^ ratio was found in the positive quadrant on the *y*-axis. Moreover, the 75% NH_4_^+/^25% NO_3_^−^, 100% NH_4_^+/^0% NO_3_^−^ and 25% NH_4_^+/^75% NO_3_^−^ ratios were found in the negative quadrant on the *y*-axis. Additionally, the 0% NH_4_^+/^100% NO_3_^−^ and 50% NH_4_^+/^50% NO_3_^−^ ratios were found in the positive quadrant on the *y*-axis in the PCA analysis of lettuce grown in the hydroponic system. It showed that the control was completely different from the various applied NH_4_^+^/NO_3_^−^ ratios (100:0, 75:25, 50:50, 25:75 and 0:100%). The 100% NH_4_^+/^0% NO_3_^−^, 75% NH_4_^+/^25% NO_3_^−^ and 25% NH_4_^+/^75% NO_3_^−^ ratios did not show a significant difference from the other applied NH_4_^+^/NO_3_^−^ ratios (50:50 and 0:100%) but these treatments showed a significant difference from 50% NH_4_^+/^50% NO_3_^−^ and 0% NH_4_^+/^100% NO_3_^−^ ratios in hydroponically grown lettuce. A PLS-DA of lettuce grown in the soil cultivation system is shown (Figure 7D). The 100% NH_4_^+/^0% NO_3_^−^ and 0% NH_4_^+/^100% NO_3_^−^ ratios were observed to result in a significant difference in metabolic profile of lettuce and were found in the positive and negative quadrants, respectively, as compared to the other applied NH_4_^+^/NO_3_^−^ ratios (75:25, 50:50 and 25:75) on the *x*-axis in soil-grown lettuce. The 75% NH_4_^+/^25% NO_3_^−^ and 0% NH_4_^+/^100% NO_3_^−^ ratios were observed in the negative quadrant on the *y*-axis, while the other applied NH_4_^+^/NO_3_^−^ ratios (100:0, 50:50, 25:75%) were found in the positive quadrant on the *y*-axis in soil-grown lettuce. A PLS-DA of lettuce grown in the substrate system is shown (Figure 7E). The applied NH_4_^+^/NO_3_^−^ ratios (100:0, 75:25, 50:50, 25:75 and 0:100%) did not show a significant difference in the metabolomic profiling of lettuce. However, the control treatment showed a significant difference in the metabolomic profile as compared to the applied NH_4_^+^/NO_3_^−^ ratios in substrate-grown lettuce. The metabolomic profiles of lettuce with the applied NH_4_^+^/NO_3_^−^ ratios (100:0, 75:25, 50:50, 25:75 and 0:100%) were found in the positive quadrant as compared to the control on the *y*-axis in the PLS-DA of substrate grown lettuce. For the lettuce grown in the hydroponic system, the control treatment was found in the negative quadrant on the *y*-axis in the PLS-DA analysis. In addition, the other applied NH_4_^+^/NO_3_^−^ ratios (100:0, 75:25, 50:50, 25:75 and 0:100%) were found in the positive quadrant on the *y*-axis in the PLS-DA of lettuce grown in the hydroponic system analyzed by GC-MS (Figure 7F).

**Figure 8:**
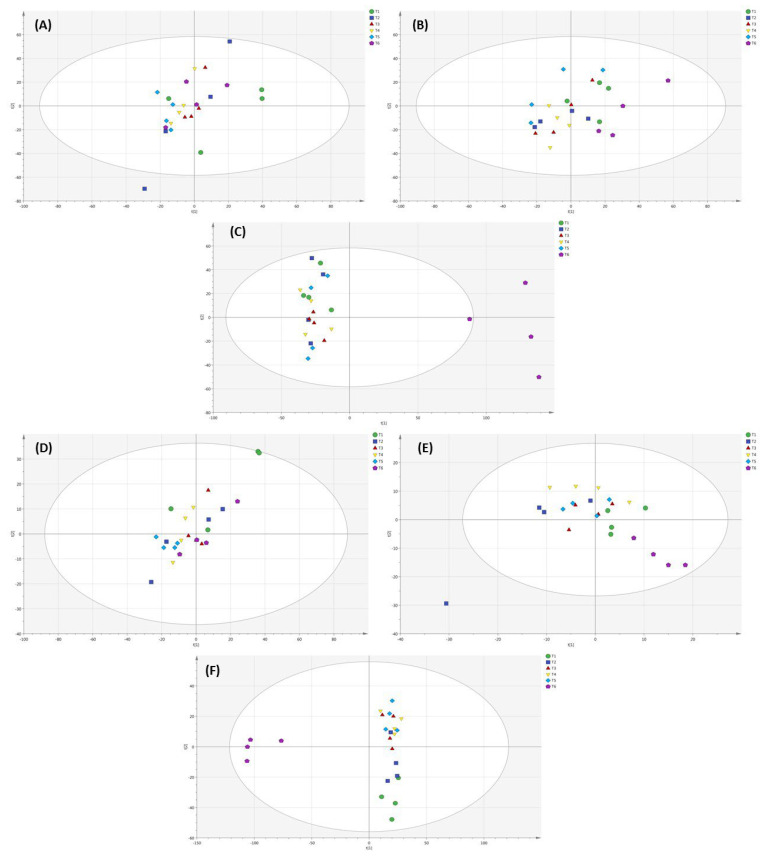
PCA and PLS-DA analysis of the metabolomic profile of lettuce with different applied NH_4_^+^/NO_3_^−^ ratios (100:0, 75:25, 50:50, 25:75 and 0:100%) and control (no additional N applied) in soil, substrate and hydroponic cultivation systems by UPLC VION IMS QTOF-MS/MS analysis. Note- (**A**–**C**) represents PCA analysis and (**D**–**F**) represents PLS-DA analysis of metabolomic profiling of lettuce in soil, substrate and hydroponic cultivation system by UPLC VION IMS QTOF-MS/MS analysis. Green color represents NH_4_^+^/NO_3_^−^ (100/0%), blue color represents NH_4_^+^/NO_3_^−^ (75/25%), red color represents NH_4_^+^/NO_3_^−^ (50/50%), yellow color represents NH_4_^+^/NO_3_^−^ (25/75%), sky-blue color represents NH_4_^+^/NO_3_^−^ (0/100%) and purple color represents control.

In PCA analysis of lettuce analyzed by UPLC VION IMS QTOF-MS/MS (Figure 8A), the 100% NH_4_^+/^0% NO_3_^−^ ratio showed a significant difference in the metabolomic profile of lettuce as compared to the other applied NH_4_^+^/NO_3_^−^ ratios in soil-grown lettuce. The metabolomic profile of lettuce showed a significant difference among the applied NH_4_^+^/NO_3_^−^ ratios and the control in substrate- and hydroponically grown lettuce (Figure 8B,C). In PCA and PLS-DA analysis of lettuce through UPLC VION IMS QTOF-MS/MS, the metabolomic profile of lettuce did not show a clear difference among the applied NH_4_^+^/NO_3_^−^ ratios in soil-grown lettuce (Figure 8A,D), while the applied NH_4_^+^/NO_3_^−^ ratios and the control showed a significant difference in metabolomic profile of lettuce analyzed by PCA (Figure 8B,C) and PLS-DA (Figure 8E,F) in substrate- and hydroponically grown lettuce.

**Figure 9:**
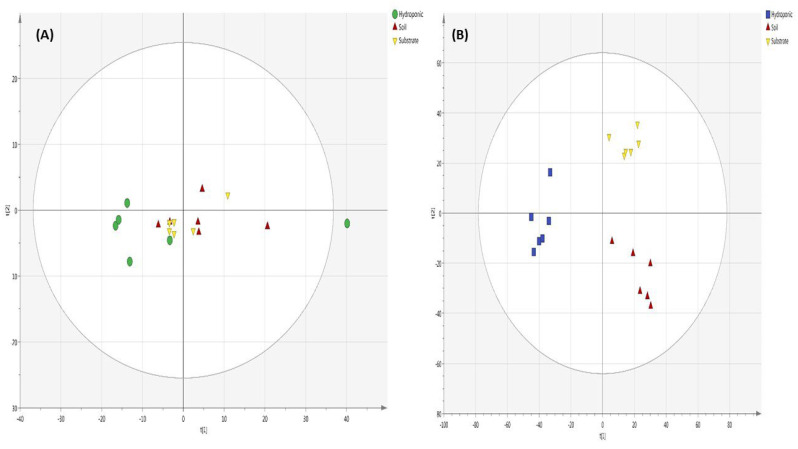
PLS-DA analysis of whole metabolomic profiling of lettuce through GC-MS and UPLC VION IMS QTOF-MS/MS analysis by combining means of all different applied NH_4_^+^/NO_3_^−^ ratios (100:0, 75:25, 50:50, 25:75 and 0:100%) and control (no additional N applied) in soil, substrate and hydroponic cultivation systems. Note- (**A**) PLS-DA analysis using GC-MS in which green color represents hydroponically grown lettuce, red color represents soil-grown lettuce and yellow color represents substrate-grown lettuce; (**B**) PLS-DA analysis using UPLC VION IMS QTOF-MS/MS in which blue color represents hydroponically grown lettuce, red color represents soil-grown lettuce and yellow color represents substrate-grown lettuce.

**Figure 10:**
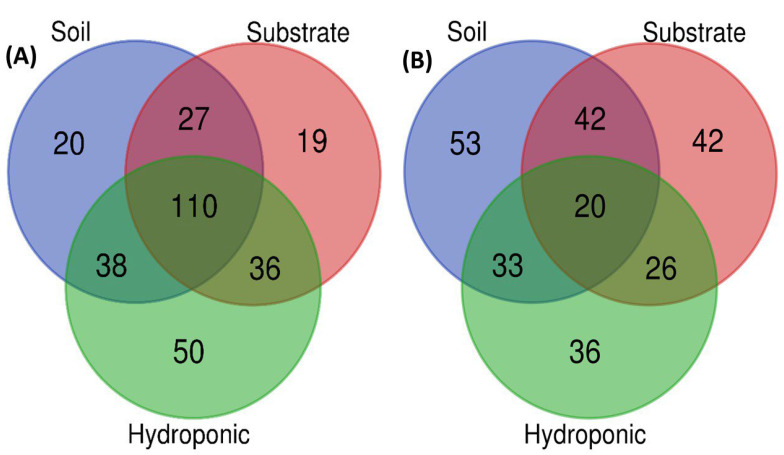
Venn diagrams of lettuce metabolites in soil, substrate and hydroponic cultivation systems using GC-MS and UPLC VION IMS QTOF-MS/MS analysis. Note- (**A**) Similar and different metabolites in metabolomic profiling of lettuce in soil, substrate and hydroponic cultivation systems using GC-MS analysis; (**B**) similar and different metabolites in metabolomic profiles of lettuce in soil, substrate and hydroponic cultivation systems using UPLC VION IMS QTOF-MS/MS analysis.

In PLS-DA, the metabolomic profiling of lettuce through GC-MS showed that soil- and substrate-grown lettuce did not show a significant difference (Figure 9A). The lettuce grown in the hydroponic system showed a significant difference in the metabolomic profile of lettuce as compared to soil and substrate systems, irrespective of the applied NH_4_^+^/NO_3_^−^ ratios and control treatments. However, metabolomic profiling of lettuce analyzed by UPLC VION IMS QTOF-MS/MS showed a significant difference among the soil, substrate and hydroponic cultivation systems, in the metabolomic profile of lettuce, irrespective of the applied NH_4_^+^/NO_3_^−^ (100:0, 75:25, 50:50, 25:75 and 0:100%) treatment (Figure 9B). Common metabolites among soil-, substrate- and hydroponically grown lettuce are shown in a Venn diagram (Figure 10A,B). In Figure 10A,B, whole metabolomic profile of lettuce grown in soil, substrate and hydroponic cultivation systems through GC-MS and UPLC VION IMS QTOF-MS/MS is presented, respectively. It was observed that 110 of the similar metabolites were found in soil-, substrate- and hydroponically grown lettuce. Lettuce cultivated in soil and substrate systems contained 20 and 19 different metabolomic compositions, respectively. The lettuce grown in the hydroponic system contained 50 different metabolites, as determined by GC-MS. In addition, 53, 42 and 36 different types of metabolic compounds were observed in lettuce using UPLC VION IMS QTOF-MS/MS in soil-, substrate- and hydroponic systems, respectively. Thus, it showed that lettuce grown in soil, substrate and hydroponic systems contained different metabolic compositions, irrespective of the applied NH_4_^+^/NO_3_^−^ ratios. The overall, discrimination of metabolic profiles of lettuce can be found in the Supplementary Data (Figures S1–S4).

**Figure 11:**
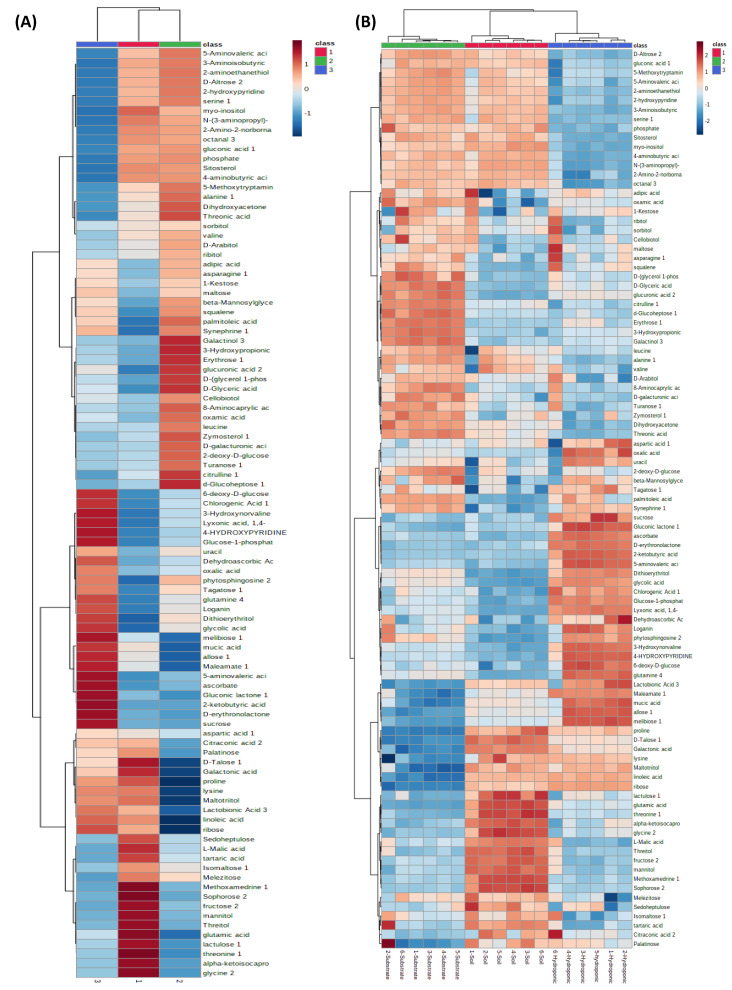
Heat map of different metabolites in soil-, substrate- and hydroponically grown lettuce. Note- Left-side (**A**) shows up- and down-regulation of metabolites in metabolomic profiles of lettuce in soil, substrate and hydroponic cultivation systems by combining means of all different applied NH_4_^+^/NO_3_^−^ ratios (100:0, 75:25, 50:50, 25:75 and 0:100%) and control (no additional N applied) in hydroponic, soil, and substrate cultivation systems. Right-side (**B**) shows variation in up- and down-regulation of metabolites in metabolomic profiles of lettuce by various applied NH_4_^+^/NO_3_^−^ ratios (100:0, 75:25, 50:50, 25:7 and, 0:100%) and control (no additional N applied) in hydroponic, soil, and substrate cultivation systems. Red color represents soil-grown lettuce metabolites, green color represents substrate-grown lettuce metabolites and blue color represents hydroponically grown lettuce metabolites.

A heat map of different metabolites in soil-, substrate- and hydroponically grown lettuce is presented in Figure 11A,B. Figure 11 shows that sophorose, fructose, mannitol, threitol, glutamin acid, lactulose, thronine and glycine were up-regulated in the soil-grown lettuce as compared to the substrate- and hydroponically grown lettuce. 5-Aminovaleric acid, D-Altrose, serine, 2-hydroxypyridine, dihydroxy acetone, threonic acid and alanine were found to be up-regulated in substrate-grown lettuce as compared to lettuce grown in soil and hydroponic systems. However, chlorogenic acid, 6-deoxy-D-glucose, d-glucoheptose, maleamate, ascorbate, gluconic lactone, 2-ketobutyric acid and sucrose were found to be up-regulated in hydroponically grown lettuce as compared to soil and substrate systems. In addition, erythrose, 3-hydroxypropionic acid and galactinol were observed to be up-regulated with all applied NH_4_^+^/NO_3_^−^ ratios in substrate-grown lettuce as compared to soil and hydroponic systems (Figure 11B). However, ascorbate and gluconic lactone were found to be up-regulated with all applied NH_4_^+^/NO_3_^−^ ratios in hydroponically grown lettuce as compared to soil and substrate systems. In lettuce grown in the hydroponic system, chlorogenic acid was found at a higher level and was upregulated in the control as compared to soil and substrate systems. This suggests that the applied NH_4_^+^/NO_3_^−^ ratios showed less influence on chlorogenic acid in hydroponically grown lettuce. Glutamine and loganin were influenced by the applied NH_4_^+^/NO_3_^−^ ratios, and were upregulated in the hydroponically grown lettuce by all applied NH_4_^+^/NO_3_^−^ ratios as compared to the control. In lettuce grown in the soil system, glutamic acid, thronine, methoxamedrine and mannitol influenced with the 25% NH_4_^+^/75% NO_3_^−^ ratio and up-regulated as compared to the 100% NH_4_^+^/0% NO_3_^−^ ratio. Higher levels of adipic acid and oxamic acid were found for the 100% NH_4_^+^/0% NO_3_^−^ ratio and upregulated as compared to the other applied NH_4_^+^/NO_3_^−^ ratios and the control in soil-grown lettuce.

## 3. Discussion

Different NH_4_^+^/NO_3_^−^ ratios have different effects on different plants under different growing conditions, probably due to the uptake and preference of N absorption from different sources and types [7,11]. In this experiment, the 25% NH_4_^+/^75% NO_3_^−^ ratio was found to be more beneficial for the physiological growth of lettuce and resulted in higher relative chlorophyll contents in soil-, substrate- and hydroponically grown lettuce. The maximum relative chlorophyll contents of lettuce were observed with the 25% NH_4_^+/^75% NO_3_^−^ ratio in the hydroponic system as compared to soil and substrate systems. It has been reported in previous studies that hydroponically grown lettuce showed an increase in physiological growth compared to soil-grown lettuce [25]. This higher growth in the hydroponic cultivation system might be due to the higher acquisition of nutrients. Different forms of N in appropriate ratios influence the chlorophyll contents. Previously, it has been reported that the application of only the NO_3_^−^ form of N reduced the chlorophyll contents. Our results are in accordance with previous studies that reported that the relative chlorophyll contents were significantly improved by the 25% NH_4_^+/^75% NO_3_^−^ ratio [40,41,42]. The application of NH_4_^+^/NO_3_^−^ ratios influenced chlorophyll synthesis and ultimately increased the chlorophyll contents of the plant [42]. The same trend has been observed with the 25% NH_4_^+/^75% NO_3_^−^ ratio in spinach [43], Chinese cabbage [42] and pepper [44].

The chlorophyll synthesis is associated with different N sources, which are ultimately correlated with the photosynthesis rate and determine the final crop yield [41]. The application of NH_4_^+^/NO_3_^−^ ratios increases the surface area of the plant by increasing the photosynthesis rate and ultimately increases the plant biomass. In this experiment, the applied 25% NH_4_^+/^75% NO_3_^−^ ratio increased the shoot fresh biomass of lettuce in soil, substrate and hydroponic cultivation systems. In the early stages of plant growth (7–14 days), the applied 25% NH_4_^+/^75% NO_3_^−^ ratio did not show a significant difference in the shoot fresh biomass of lettuce as compared to other the applied NH_4_^+^/NO_3_^−^ ratios in soil, substrate and hydroponic systems. This might be due to the lower amount of N requirement in the early stages of plant growth. However, in the later stages of plant growth (28–49 days), a consistent increase in shoot fresh biomass was observed with the 25% NH_4_^+/^75% NO_3_^−^ ratio in lettuce grown in soil, substrate and hydroponic systems. This might be due to the higher consumption of N by plants. During this period, a consistent increase in shoot fresh biomass of lettuce was observed with the applied 25% NH_4_^+/^75% NO_3_^−^ ratio and an increase of >30% in lettuce fresh biomass as compared to the control and other applied NH_4_^+^/NO_3_^−^ ratios in lettuce grown in the three cultivation systems. Some studies have been reported that the fresh biomass of canola was increased with the 25% NH_4_^+/^75% NO_3_^−^ ratio [45] and 45% NH_4_^+/^55% NO_3_^−^ ratio in *Panicum* [46]. A similar trend has been observed in spinach [43], Chinese cabbage [9,42], and pepper [43]. The higher translocation of N from the xylem to the leaves could be one of the reasons behind this [47].

Taste and quality parameters are two important factors of plants that are greatly influenced by the applied NH_4_^+^/NO_3_^−^ ratio and are responsible for the sensory acceptance of lettuce. In our study, the relative abundance of ascorbate, glutamic acid and lactupicrin, as taste and quality-related compounds in lettuce, was determined. Our study showed that the relative abundance of ascorbate increased with the 25% NH_4_^+/^75% NO_3_^−^ ratio, as compared to the other applied NH_4_^+^/NO_3_^−^ ratios in substrate- and hydroponically grown lettuce. However, the 50% NH_4_^+/^50% NO_3_^−^ ratio increased the relative abundance of ascorbate in the soil-grown lettuce. This trend might be due to NH_4_^+^/NO_3_^−^ ratios that increase enzymatic activities that oxidize ascorbic acid and produce water and dehydro-ascorbic acid in plants [48]. The study has reported that in the plant process with the applied 10% NH_4_^+^/90% NO_3_^−^ ratios and the control treatments, two key enzymes are involved in controlling the reactive oxygen species (ROS) in plants and maintaining the ascorbate contents in plants. Ascorbate-mediated redox reactions with light absorption are important for improving the anthocyanin synthesis in the plants and ultimately improving the quality of the plants [49]. It has been reported that appropriate NH_4_^+^/NO_3_^−^ ratios influenced the antioxidant activities under N stress conditions [50]. Health-promoting compounds such as anthocyanin, ascorbate (vitamin C) and polyphenols in plants have been increased due to counteracting ROS in plants under N stress [51,52,53,54].

In this experiment, the relative abundance of glutamic acid in lettuce increased with the 25% NH_4_^+/^75% NO_3_^−^ ratio as compared to the other applied NH_4_^+^/NO_3_^−^ ratios and the control in soil, substrate and hydroponic cultivation systems. However, the relative abundance of lactupicrin was influenced by the 25% NH_4_^+/^75% NO_3_^−^ ratio in the soil cultivation system. The N involvement in the primary and secondary metabolism of the plants has been reported as one of the reasons that the application of N influences taste- and quality-related parameters [55,56,57,58]. It has been reported that glutamic acid increased in maize [59] and lactupicrin increased in lettuce plants [60] due to N assimilation in plants. The NH_4_^+^/NO_3_^−^ ratio plays an important role in many signaling pathways, leading to the accumulation of abundant metabolites that contributed to the diverse quality and taste of lettuce. Interestingly, these metabolites accumulate at a median level in N assimilation. In N assimilation, nitric oxide has been reported in regulation of many biological processes as a signaling molecule, including flavonoid accumulation and phosphorus re-utilization with NH_4_^+^/NO_3_^−^ ratios. It could be conceived that there was cross-talk between signaling pathways to regulate the accumulation of primary and secondary metabolites related to diverse lettuce quality and taste-related compounds with NH_4_^+^/NO_3_^−^ ratios and the control in different cultivation systems.

The requirement of primary and secondary metabolites has been reported with N application for their biosynthesis [61,62]. However, increasing the N application cannot fulfill the requirements of all metabolites in plants due to the common need for their biosynthesis [63,64,65,66]. Thus, the mixed application of different NH_4_^+^/NO_3_^−^ ratios is very important for metabolomic activities in lettuce. The application of NH_4_^+^/NO_3_^−^ ratios have been considered to be of paramount importance to reduce the environmental effect and improved the N use efficiency [67]. Plant primary and secondary metabolic contents have been reduced when applying only a single form of N or when applying it in excess without mixing the NH_4_^+^/NO_3_^−^ ratios due to internal competition for plants [68]. In the present study, sugar-related compounds (sucrose, fructose, lactulose, and maltose) in lettuce identified and analyzed via GC-MS and UPLC VION LC-MS/MS. The results showed that the relative abundance of sucrose, lactulose, and maltose in lettuce was higher with the 25% NH_4_^+/^75% NO_3_^−^ ratio as compared to the other applied NH_4_^+^/NO_3_^−^ ratios in soil, substrate and hydroponic systems. In addition, the relative abundance of fructose was increased with the 50% NH_4_^+/^50% NO_3_^−^ ratio in soil- and hydroponically grown lettuce. On the other hand, the relative abundance of fructose was increased with the 25% NH_4_^+/^75% NO_3_^−^ ratio in lettuce grown in the substrate system. It has been reported that plants produced more sugar contents with the help of NH_4_^+^/NO_3_^−^ for their survival and produced more defense-related metabolites with the 25% NH_4_^+/^75% NO_3_^−^ ratio [69]. Additionally, the NH_4_^+^/NO_3_^−^ ratios influenced different sugar-related compounds and reduced the inhibitory effects on the level of sugar compounds by balancing the applied NH_4_^+^/NO_3_^−^ ratios in lettuce grown in soil, substrate and hydroponic systems. Therefore, identifying the appropriate NH_4_^+^/NO_3_^−^ ratio is an important component of improving sugar metabolism in plants. Sugar compounds play key role in plant growth and nutritional values under N application. These results are consistent with previous studies using soybean grains [70] and sugarbeet root [71]. Sugar compounds are also one of the most significant determining factors of yield and quality in plants [72,73,74]. Sugar compounds are the main source of energy in photosynthesis and transport carbohydrates over a long-distance in plant leaves, which are responsible for better plant growth when using NH_4_^+^/NO_3_^−^ ratios [74,75,76,77].

N assimilation in plants plays an important role in plant growth, development and protein accumulation [78,79]. In our study, amino acid contents in lettuce were increased by applying 25% NH_4_^+/^75% NO_3_^−^ ratio in soil, substrate and hydroponic cultivation systems. Lettuce grown in the soil system showed a higher increase in amino acid contents as compared to the substrate and hydroponic system. Amino acid transporters are triggered to a greater extent by the applied NH_4_^+^/NO_3_^−^ ratios in the soil as compared to the hydroponic system. The amino acid profile were influenced by different N application forms, which showed a great impact on the contents of amino acids in different plant parts [80,81]. The most important function of amino acid transporters is the movement of amino acids from source to sink, i.e., plant leaves and seeds to fruits and the exertion of a regulatory mechanism for the uptake of N, the synthesis of amino acids and the allocation of amino acids in plants [79,81]. Positive feedback of N assimilation helped to increase the N uptake from the solution to roots and roots to shoots due to the applied NH_4_^+^/NO_3_^−^ ratios and ultimately increased the various types of amino acids in the soil as compared to the hydroponic cultivation system. It has been reported that some other factors also have a greater influence on amino acid formation in the soil as compared to hydroponically grown plants [82,83,84,85]. The changes in the relative abundance of amino acids and polyphenolic compounds were found to be similar to changes in NH_4_^+^ content in plants in previous studies [86,87]. This trend was found to be in accordance with the results reported by Arnold et al. [84] and it can be concluded that a higher concentration of NO_3_^−^ application stimulates N assimilation, which is used for primary and secondary metabolite synthesis in plants. In lettuce, amino acid compounds not only played a central role in N metabolism but also markedly impacted the lettuce quality, i.e., alanine, valine, leucine and proline. Concentrations of alanine, valine, leucine and proline were found to be considerably higher in soil-, substrate- and hydroponically grown lettuce treated with the 25% NH_4_^+/^75% NO_3_^−^ ratio than in lettuce treated with the other applied NH_4_^+^/NO_3_^−^ ratios. In many plants, these amino acids have represented an intermediate link in N metabolism and contributed to N transport in plant [85]. Based on these results, plants might employ a specific N re-allocation strategy to acclimate to applied NH_4_^+^/NO_3_^−^ ratios in all three cultivation systems. The amino acid contents (alanine, valine, leucine, serine, lysine and proline) were also reduced with the control treatment and were increased with the applied NH_4_^+^/NO_3_^−^ ratios in soil-, substrate- and hydroponically grown lettuce. These amino acid contents may be involved in the formation of oxalacetate, an important intermediate in the tricarboxylic acid cycle (TCA); the assimilation of NH_4_^+^ into amides and amino acids requires carbon skeletons from the TCA [86]. The TCA cycle could increase the energy for amino acid synthesis [79] and thereby enhances the contents of protein and amino acids. Most amino acids are primarily related to N storage and utilization [87,88]. Amino acids are downstream products of N metabolism and their abundance increases with elevated balanced N applications [89,90]. Overall, lower amino acid synthesis rates in plants as compared to the mixture of NH_4_^+^/NO_3_^−^ ratios have stimulated N assimilation in plants [9].

The N application also showed a significant effect on secondary metabolites biosynthesis in the plants. Different polyphenols (1-O-caffeoylglucose, 1,3-dicaffeoylquinic acid, quercetin-3-galactoside, naringin 6’’-rhamnoside, aesculetin, viscutin 1, sinaticin and (+)-myristinin A) were analyzed during the experiment. The results revealed that all polyphenols showed an increasing trend in their relative abundance in lettuce, while the maximum relative abundance of all of the above-mentioned polyphenols in lettuce was observed in the control in the substrate and hydroponic systems. The applied 100% NH_4_^+/^0% NO_3_^−^ ratio increased the relative abundance of polyphenols in the soil-grown lettuce. It has been reported that 14% of total flavonoids were influenced by the control application in tomatoes [82]. Naringin and rutinoside flavonoids in fruits have been influenced with N applications and the control [91]. Total flavonoids and chlorogenic acids in apple skins and secondary metabolites in apple leaves have been altered with N applications and the control [72,73]. It has been reported that N influenced the flavonoids and antioxidants in *Axonopus compressus* and *Coreopsis tinctoria* Nutt [89,90,91]. The increase in flavonoids under different N fertilization treatments might be attributed to an increase in phenylalanine availability due to the restriction of protein synthesis under N deficiency. The phenylalanine availability could be substantially enhanced the production of secondary metabolites (polyphenols) as phenylalanine has been reported as a precursor for the formation of flavonoids in plants [92,93].

The overall metabolic profiles of lettuce grown in soil, substrate and hydroponic cultivation systems were investigated based on an untargeted metabolomics approach. PCA analysis showed that the applied NH_4_^+^/NO_3_^−^ ratios (100:0, 75:25, 50:50, 25:75 and 0:100%) and control showed no clear separation into four clusters for substrate-grown lettuce metabolites. Additionally, the 100% NH_4_^+/^0% NO_3_^−^ ratio showed a significant difference in metabolites in substrate-grown lettuce. However, a significant difference in lettuce metabolites with the control treatment was observed via PLS-DA analysis in hydroponic system. Under the control treatment, similar clustering has been observed in traditional Chinese herb (*Isatis indigotica*) [53] and tea plants, which showed more changes in their metabolomic profiles of plants as compared to N treatments according to OPLS-DA analysis [94]. Under the applied NH_4_^+^/NO_3_^−^ ratio and the control treatments, NH_4_^+^ and NO_3_^−^ assimilation has produced nitric oxide, which influences reactive oxygen species (ROS) in plants and ultimately changes the metabolic changes in plants. A similar finding was observed in the leaves of tomato plants [54]. In our study, a clear separation of the cluster of lettuce metabolites was shown (Figure 9 and Figure 10), analyzed using GC-MS and UPLC VION IMS QTOF-MS/MS analysis in soil, substrate and hydroponic systems. We found a clear difference in the metabolomic profiles of lettuce in soil, substrate and hydroponic systems. These results demonstrate that the applied NH_4_^+^/NO_3_^−^ ratios and control treatment in different cultivation systems influenced the metabolomic profiles of lettuce but it depended on the growing condition of lettuce and nitrogen assimilation in the lettuce-grown cultivation systems.

Thus, it can be concluded that the uptake of the mixture of NH_4_^+^/NO_3_^−^ ratios for improving the parameters of the above-mentioned taste- and quality-promoting compounds, sugar-related compounds, amino acid contents and polyphenolic compounds significantly increased the growth, development and nutritional value of lettuce.

## 4. Materials and Methods

A pot experiment was conducted in a greenhouse at the School of Agriculture and Biology, Shanghai Jiao Tong University, Shanghai to evaluate the influence of the ammonium to nitrate ratio on plant growth, sugar compounds, amino acid contents and polyphenolic compounds in lettuce in soil, substrate and hydroponic cultivation systems. For this purpose, loose-leaf type lettuce (*Lactuca sativa* L. cv. Yidali) was cultivated in soil, substrate and hydroponic systems for 49 days. For pre-sowing analysis, composite samples of soil and substrate were collected and analyzed for physicochemical properties as shown in Table 1 [95,96].

Lettuce seeds were grown for germination in a tray at 18–22 °C temperature and 60% relative humidity with 12 h in light and 12 h in dark. After 2 weeks, two leaves of lettuce plants were shifted to pots and the hydroponic system to analyze the physiological growth and metabolite changes in lettuce. Healthy plants with two leaves were shifted in each pot in soil and substrate cultivation systems. Then, six different treatments including control (no additional N applied) and five different NH_4_^+^/NO_3_^−^ ratios (100:0, 75:25, 50:50, 25:75 and 0:100%) were applied to the two-leaf stage plants with 4 replications. About 20% N was applied to the base of the soil before sowing.

Table 2 shows the applied NH_4_^+^/NO_3_^−^ ratios and other nutrient solutions in the hydroponic cultivation system [40]. In addition, inhibitor dicyanamide was added to the nutrient solution to inhibit nitrification. For environmental conditions during the experiment, day-time and night-time temperatures were 22 ± 2 °C and 16 ± 2 °C, respectively with a photoperiod of 12 h ranging from 240–290 µmol m^−2^s^−1^ during the whole experiment. Ammonium sulfate was used for the NH_4_^+^ source while calcium nitrate was used as NO_3_^−^ to make a ratio. The nutrient solution was renewed every three days with proper aeration in the hydroponic system and the pH of the solution was adjusted between 5.8–6.5 with 0.5 mol L^−1^ NaOH or HCL.

Plants were harvested after 7 days of N application in soil-, substrate- and hydroponically grown lettuce. Plant samples were collected 7 times in total (7–49 days after N application). Fresh biomass of shoots was determined periodically from 7 days to 49 days using an electronic balance.

After 21 days, Dualex 4 Scientific^™^ (FORCE-A, Orsay, France) was used to measure the relative chlorophyll contents. Readings were taken from the upper tip, middle part and lower part of lettuce from 4 different leaves of one plant in one replication and this process was repeated for all treatments in three cultivation systems [97].

For metabolomic analysis, gas chromatography mass spectrometry (GC-MS) and ultra-performance liquid chromatography–quadrupole-time of flight-mass spectrometry (UPLC VION IMS QTOF-MS/MS) was used. A total of 24 plants were collected by applying 6 different treatments with 4 replications from one cultivation system. In this way, a total of 72 lettuce plants were collected from lettuce grown in soil, substrate and hydroponic cultivation systems after 49 days. All samples were stored after being dipped in liquid N and frozen at −80 °C for further analysis.

### 4.1. Chemicals and Reagents

The chemical reagents, bis-(trimethylsilyl)-trifluoroacetamide (BSTFA) and saturated alkane standards (C7–C40) were purchased from Sigma Aldrich Trading Co., Ltd. (Shanghai, China). Chloroform was purchased from Shanghai Titan Technology Co., Ltd. (Shanghai, China). Sulfuric acid was purchased from the Sinopharm Chemical Reagent Co., Ltd. (Shanghai, China). Trimethylchlorosilane, pyridine and methoxyamine hydrochloride were purchased from Myrell Chemical Technology Co., Ltd. (Shanghai, China). L-2-Chlorophenylalanine was purchased from Shanghai Bi De Pharmaceutical Technology Co., Ltd. (Shanghai, China). Quercetin 3-β-D-glucoside was purchased from Macklin Shanghai Macleans Biochemical Technology Co., Ltd. (Shanghai, China). Methanol and acetonitrile were purchased from Shanghai Aladdin Biochemical Technology Co., Ltd. (Shanghai, China). Milli-Q system (Millipore, Bedford, MA, USA) was used to obtain ultrapure water for analysis. All purchased chemicals were HPLC grade ≥ 98%.

### 4.2. Untargeted Metabolomic Analysis through GC-MS and UPLC VION IMS QTOF-MS/MS

#### 4.2.1. Sample Preparation for GC-MS and UPLC VION IMS QTOF-MS/MS

The extraction protocol for GC-MS was modified by Du et al. [98]. Briefly, 300 mg lettuce leaf samples were ground and frozen in liquid N. A mixture of chloroform and methanol (1:3 *v*/*v*) was vortexed at 6000 rpm for 15 s (repeated 3 times) and used for extraction. L-2-chlorophenylalanine was used as an internal standard and added to the samples (20 μL of 0.3 mg mL^−1^). The samples were centrifuged at 12,000 g for 15 min (min) in a centrifuge machine (CT15RE, Hitachi, Tokyo, Japan). After centrifuging, the supernatant of the sample (0.3 mL) was transferred to a tube. To dry the samples, a Speed-Vac coupled with a cold trap was used at room temperature for 2 h (h). For derivation, methoxyamine hydrochloride (80 μL) was used in the dried extract with pyridine (20 mg mL^−1^) and incubated at 37 °C for 2 h. BSTFA (80 μL of 10 mg mL^−1^ trimethylchlorosilane) was added to the samples and incubated at 70 °C for 1 h.

Lettuce samples (300 mg) in liquid N were ground and added to 1 mL of water and methanol (20:80 *v*/*v*) for UPLC VION IMS QTOF-MS/MS extraction followed by sonication at 25 °C for 35 min and placed at 4 °C for 12 h. The samples were centrifuged at 12,000 g for 15 min. After centrifuging, the supernatant of the samples (0.5 mL) was transferred to a vial for further analysis [99].

#### 4.2.2. Analysis Using GC-MS and UPLC VION IMS QTOF-MS/MS

The Agilent 7890 gas chromatograph (GC) coupled with a LECO mass spectrometer (MS) (PerkinElmer Inc., Waltham, MA, USA) was used to analyze the derivative extraction samples. A DB-5MS capillary column (length (30 m) × inner diameter (0.25) mm, film thickness (0.25 μm)) (Agilent J&W Scientific, Folsom, CA, USA) was used to inject the samples (1 μL). The inlet temperature was set at 280 °C. The oven temperature for gas chromatography was maintained at 60 °C after solvent delay (6.5 min). After 1 min, the samples were injected and the temperature (300 °C) of the gas chromatography oven was increased. The oven temperature was increased by 5 °C per minute and maintained at 300 °C for 11 min. The temperature of the transfer ion and line source was set at 230 °C and 280 °C, respectively. Helium gas was used as a carrier gas with a 1 mL min^−1^ flow rate. In full scan mode from 33 to 600 m/z, electron impact ionization (70 eV) was measured. The saturated alkane standards (C7–C40) were injected at 1 μL (10 μg·mL^−1^ final concentration) and analyzed before sample analysis.

The Acquity UPLC VION IMS QTOF-MS/MS (Waters Corp., Milford, MA, USA) was used to determine the untargeted metabolomics of lettuce on the basis of MS/MS data, retention time, mass/charge ratio and collision cross-section (CCS). An Acquity UPLC HSS T3 column (100 mm × 2.1 mm, 1.7 μm; Waters Corp.) was used to inject the sample (3 μL) for analytical conditions. The temperature was set at 40 °C. In the mobile phase, water and acetonitrile were maintained at 0.4 mL per minute for 0 to 3 min with 100% gradient elution. Water with a 90 to 100% linear gradient was maintained for 3 to 5 min. Water with a 65–90% linear gradient was maintained for 5 to 11.5 min. Water with a 1–65% linear gradient was maintained for 11.5 to 14 min; 1% linear gradient was maintained for 14 to 17 min and 1 to 100% linear gradient was maintained for 17 to 17.1 min with water. Finally, the required conditions were attained and equilibrium of the column was maintained for 5 min. Capillary voltage of 2 kV was used at 115 °C and 450 °C for source temperature and desolvation temperature, respectively, with 800 L per hour for the desolvation gas flow. The collision energy was 20 to 55 eV, and spectra were obtained with a 50 to 1000 m/z scan range in negative ion mode. Data-dependent acquisition (DDA) and data-independent acquisition (HDMSe) were performed in ion mobility for MS scanning. Quality control (QC) samples were analyzed to assess the analytical performance of the samples by mixing aliquots of all samples. The QC samples were analyzed and injected at the beginning, middle and end of each batch to assess the characteristics of mass spectrometry and chromatography (mass accuracy, signal intensity, CCS value and retention time stability).

The UPLC VION IMS QTOF-MS/MS and GC-MS raw data were used for filtering, deconvolution, alignment and normalization by using UNIFI (Waters, Corp., Milford, MA, USA) and LECO Chroma TOF (PerkinElmer Inc., Waltham, MI, USA), respectively. The processed UPLC VION IMS QTOF-MS/MS and GC-MS data were used for the analysis of differences among the applied treatments under different cultivation systems by exporting data in Progenesis QI (Waters, Corp. MA, USA) and SIMCA-14.1 (Umetrics, Umeå, Sweden) for unsupervised principal analysis (PCA) and supervised partial least squares discriminant analysis (PLS-DA) segregation. In multivariate analysis, variable importance projection (VIP) values > 1 and *p*-value < 0.05 (HSD Tukey’s test) were used to identify metabolites from GC-MS and UPLC VION IMS QTOF-MS/MS in the soil, substrate and hydroponically grown lettuce. The complete information about metabolomic profiling discrimination in lettuce can be found in the Supplementary Data (Figures S1–S4). However, significant metabolites obtained with different applied NH_4_^+^/NO_3_^−^ ratios and the control in soil-, substrate- and hydroponically grown lettuce were selected and discussed. In GC-MS, candidate metabolites were identified by using LECO Chroma TOF through comparison with reference spectra in NIST 2014 mass spectral database. All matched mass spectral threshold levels (200 to 1000) and retention index deviations (RI index) less than 1% were used for manual supervision. The peak area was determined by using the selected mass quantification of each metabolite and the relative abundance of each metabolite was determined by dividing the peak area of the candidate metabolite by the peak area of internal standard (L-2-chlorophenylalanine).

The candidate metabolite identification from processed through UPLC VION IMS QTOF-MS/MS data was performed by using the CCS values, MS2 fragment isotopic distribution, retention time and accurate mass by comparison with online databases (HMDB, Lipidmaps, Metlin and ReSpect), published bibliographies and inhouse database (Waters integrated natural product). For database comparison, CCS values with acceptable error (5%), MS tolerance (3 mDa) and MS/MS tolerance (10 mDa) were used. The preliminarily identified compounds were verified by searching in KEGG and PlantCyc. Thus, identified candidate metabolites were resolved in the chromatograms by using standards and comparing them with inhouse and online databases as well as bibliographies related to lettuce metabolites via spectral features (CCS values, MS2 fragment isotopic distribution and accurate masses). When not available, CCS values were predicted by means of our CCS prediction model [100] aimed at providing additional identification confidence.

### 4.3. Statistical Analysis

Two-way ANOVA was performed on all data by using SPSS Statistics 26.0 software (SPSS, Inc., Chicago, IL, USA). The significance of mean differences among applied treatments in soil, substrate and hydroponically grown lettuce was analyzed by Tukey’s HSD test (*p* < 0.05). All figures were created by using R software (R-4.1.2). The metabolites were exhaustively contrasted by adopting heat map and partial least squares discriminant analysis (PLS-DA) methods for the applied treatments in lettuce grown in soil, substrate and hydroponic systems. PCA and PLS-DA analyses were performed by SIMCA-14.1 (Umetrics, Umeå, Sweden) and the heatmap was generated in metaboAnalyst 5.0 (www.metaboanalyst.ca).

## 5. Conclusions

In this study, the 25% NH_4_^+/^75% NO_3_^−^ ratio promoted the relative chlorophyll contents, shoot fresh biomass, sugar-related compounds, amino acid contents, ascorbate and glutamic acid in soil, substrate and hydroponically grown lettuce. However, the relative abundance of lactupicrin, 1-O-caffeoylglucose, (+)-myristinin A, 1,3-dicaffeoylquinic acid, aesculetin, sinaticin and quercetin-3-galactoside increased with the 0% NH_4_^+/^100% NO_3_^−^ ratio in soil grown lettuce. The relative abundance of lactupicrin, 1-O-caffeoylglucose, 1,3-dicaffeoylquinic acid, aesculetin and quercetin-3-galactoside showed the opposite response to applied NH_4_^+^/NO_3_^−^ ratios in the substrate and hydroponically grown lettuce. The metabolic profile of lettuce was also influenced by appropriate NH_4_^+^/NO_3_^−^ ratios in soil, substrate and hydroponic systems, but it depended upon the desired metabolites and the cultivation system. The results showed that the 25% NH_4_^+/^75% NO_3_^−^ ratio is found more suitable for obtaining the desired physiological growth, biomass, sugar-related compounds, amino acid contents and quality- and taste-promoting compounds in soil, substrate and hydroponically grown lettuce. Among cultivation systems, hydroponically grown lettuce was found to be more suitable for higher lettuce biomass yield and polyphenolic compounds. Furthermore, soil- and substrate-grown lettuce is considered more suitable for higher amino acid contents and sugar-related compounds. These findings suggest that applied NH_4_^+^/NO_3_^−^ ratios produce different types of metabolites in soil-, substrate- and hydroponically grown lettuce and can be used as potential metabolic markers to improve the N use efficiency in lettuce. This study will be helpful for growers and the researchers for defining the NH_4_^+^/NO_3_^−^ ratios for lettuce in different cultivation systems.

## Figures and Tables

**Table 1:** Physicochemical properties of soil and substrate cultivation systems.

Parameters (Units)	Soil Values	Substrate Values
pH	6.5 ± 0.02	5.7 ± 0.04
ECe (dS m^−1^)	1.54 ± 0.23	0.83 ± 0.19
Organic matter content (g kg^−1^)	33.5 ± 0.43	488.3 ± 1.14
Total nitrogen (g 100g^−1^)	0.19 ± 0.11	0.33 ± 0.05
Ammonium (mg kg^−1^)	42.8 ± 0.29	38 ± 0.12
Nitrate (mg kg^−1^)	225.9 ± 1.43	379.5 ± 1.03
Potassium (mg kg^−1^)	22530 ± 20.67	3512 ± 5.82
Phosphorus (mg kg^−1^)	1297 ± 15.76	260.2 ± 1.35
Calcium (mg kg^−1^)	9332 ± 35.05	6528 ± 9.41
Magnesium (mg kg^−1^)	8411 ± 12.24	6274 ± 15.65
Textural class	Silt Loam	
Sand (%)	11.91 ± 0.90	
Clay (%)	12.67 ± 0.55	
Silt (%)	75.42 ± 0.81	

Note-The means (*n* = 4) ± standard errors are shown in the data.

**Table 2:** Nutrient application in soil, substrate and hydroponic cultivation systems.

NH_4_^+^/NO_3_^−^	100/0%	75/25%	50/50%	25/75%	0/100%	Control
Soil (mg Kg^−1^)	306.6/0	229.95/35.32	153.3/70.65	76.65/105.97	0/141.3	0
Hydroponic (mmol L^−1^)	5/0	3.75/1.25	2.5/2.5	1.25/3.75	5	0
Substrate (mmol L^−1^)	5/0	3.75/1.25	2.5/2.5	1.25/3.75	5	0
Other nutrients applied in hydroponic system (mmol L^−1^)						
K			1			
P			0.25			
Mg			0.36			
Ca			0.15			
Cu			0.016			
Zn			0.015			
Mn			0.01			
Fe			0.008			
B			0.002			

## Data Availability

Data is contained within the article and Supplementary Materials.

## Supplementary Materials

The following are available online at https://www.mdpi.com/article/10.3390/metabo12050444/s1, Figure S1: Different metabolites composition in soil, substrate and hydroponic cultivation system, Figure S2: Enrichment overview of top 25 of different metabolites in soil, substrate and hydroponic cultivation system, Figure S3: Top 25 of different metabolites sets in soil, substrate and hydroponic cultivation system, Figure S4: Mean decrease accuracy and VIP score of different metabolites in soil, substrate and hydroponic cultivation system.
