# Supplementary material for: Differential Metabolic Responses of Lettuce Grown in Soil, Substrate and Hydroponic Cultivation Systems under NH4+/NO3− Application
Source: Metabolites. 2022 May 16;12(5):444. doi: 10.3390/metabo12050444 (PMC9143640; doi:10.3390/metabo12050444)
Supplement: Supplementary file 1 [file metabolites-12-00444-s001.zip › metabolites-1654483-supplementary/metabolites-1654483-supplementary.pdf]

metabolites

MDPI

Differential Metabolic Responses of Lettuce Grown in Soil, Substrate and Hydroponic Cultivation Systems under Nh_4^+/No_3^- Application

The discriminant metabolites for the quality index of lettuce were screened from the GC/MS and UPLC VION IMS QTOF MS/MS metabolites under soil, substrate and hydroponic cultivation systems on the basis of VIP > 1.0 criteria for selection of metabolites (**Supplementary data 1-4**) and compared the metabolomic profiling among the soil, substrate and hydroponic cultivation systems. Higher level of fatty acids and conjugates (**Supplementary Figure 1A)** were observed in the soil as compared to substrate (**Supplementary Figure 1B)** and hydroponic cultivation systems (**Supplementary Figure 1C)** followed by amino acids and peptides while lesser level of benzenediols (Dopamine; 3,4-Dihydroxyphenylglycol) and fatty aldehydes (Octanal; Succinic acid semialdehyde) were found in the soil as compared to substrate and hydroponic cultivation systems. In substrate and hydroponic cultivation systems higher level of amino acids and peptides were found followed by fatty acids and conjugates. In substrate cultivation system, lesser level of isoprenoids compounds (Loganin and Squalene), phenols (4-Nitrophenol) and purines (Adenosine) were observed while lesser level of organic dicarboxylic acids (Glucaric acid; Malonic acid; Citraconic acid), flavonoids (Naringenin; Chrysin; Tricetin) and phenylacetic acids (3-Hydroxyphenylacetic acid; 3,4-Dihydroxymandelic acid) were observed in hydroponic cultivation system. In the pie chart**,** composition of different metabolites was presented in the soil, substrate and hydroponic cultivation systems **(Supplementary Figure 1A, B & C)**. In the soil cultivation system (**Supplementary Figure 1A)**, fatty acids and conjugated contained 2-ketobutric acid, (S)-3-Hydroxyisobutyric acid; Glycolic acid; Galactaric acid; Hydroxypropionic acid; Oxalic acid; 5-Aminopentanoic acid; 3-Aminoisobutanoic acid; Alpha-ketoisovaleric acid; Gamma-Aminobutyric acid; 2-Oxovaleric acid; Oleic acid; Palmitic acid; Adipic acid; Linoleic acid; 4-Hydroxybutyric acid; Alpha-Linolenic acid; Methyl linoleate; Maleamate; Pyruvic acid; Malonic acid; Succinic acid; Glucaric acid; Methylmalonic acid followed by amino acids and peptides (Creatine; Glycine; L-Glutamic acid; L-Tyrosine; L-Alanine; L-Proline; L-Threonine; L-Asparagine; L-Histidine; L-Lysine; L-Aspartic acid; L-Glutamine; L-Leucine; N-Acetyl-L-alanine; L-Valine; Citrulline; DL-2-Aminooctanoic acid; Guanidinosuccinic acid; N-Acetylornithine; Iminodiacetic acid; N-Acetylleucine; 5-Aminopentanamide and N-Acetyl-beta-alanine) were observed. However, enrichment overview of top 25 metabolites of lettuce grown in soil, substrate and hydroponic cultivation systems **(Supplementary Figure 2 & 3)** showed that all metabolites related to different metabolism synthesis were observed a significant difference (p value < 0.05) for different metabolites. In soil cultivation system, lettuce was observed higher in glycerophospholipid metabolism related compounds followed by steroid biosynthesis related compounds while in substrate cultivation system, alpha linolenic acid metabolomic related compounds were found in higher level followed by unsaturated fatty acids related compounds. However, nitrogen metabolism related compounds were found significantly higher in hydroponically grown lettuce as compared to lettuce grown in soil and substrate cultivation systems.

Higher accuracy value of 2-deoxy-D-glucosides (**Supplementary Figure 4A)** was found in substrate followed by hydroponic and soil cultivation systems on the basis of mean decrease accuracy while loganin was observed in lesser accuracy value in substrate cultivation system as compared to soil and hydroponic cultivation systems. In **Supplementary Figure (4B)**, Lyxonic acid, 3-hydroxynorvaline, glycolic acid, 1-hydroxypyridine, dithioerthritine and gluco-1-phosphate were found higher VIP values in hydroponic cultivation system followed by soil and substrate cultivation systems while sitosterol and 4-

aminobutyric acid were found lesser VIP values in soil cultivation system followed by hydroponic and substrate cultivation systems.

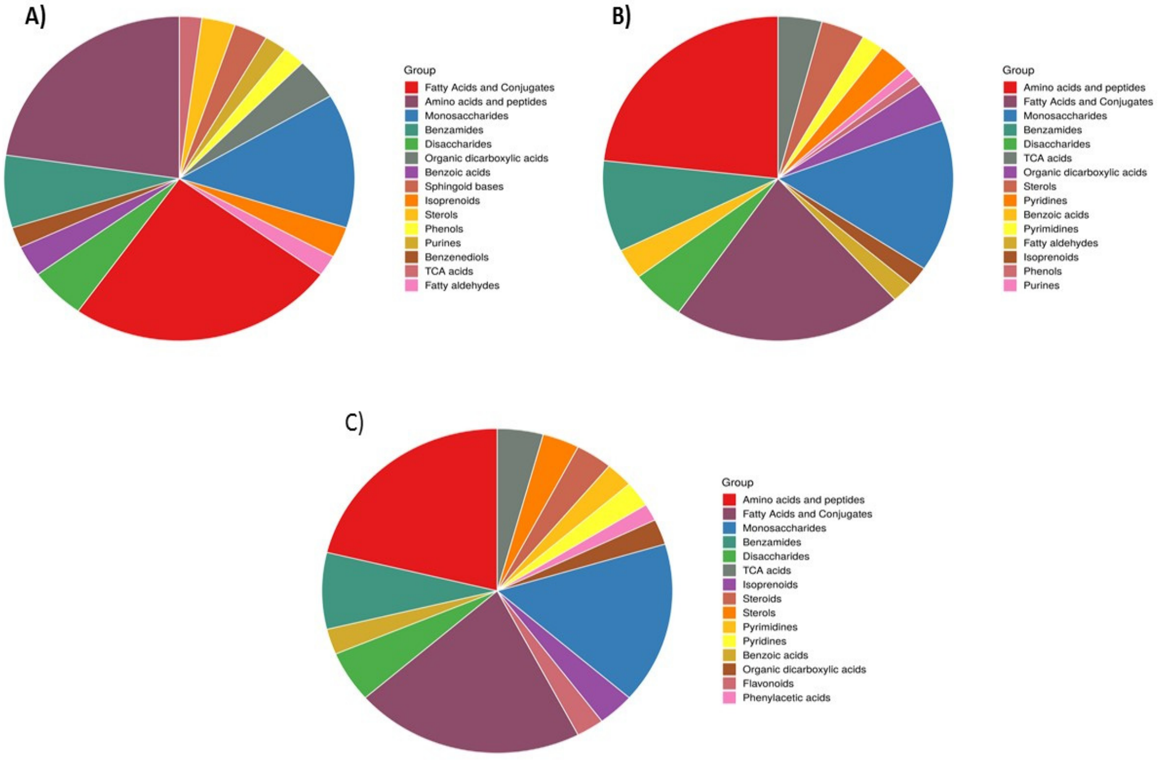

Figure S1. Different metabolites composition under soil, substrate and hydroponic cultivation system.

Note- Different metabolomic profiling of lettuce in **Figure A)** represents soil cultivation system , **Figure B)** represents substrate cultivation system and **Figure C)** represents hydroponic cultivation system

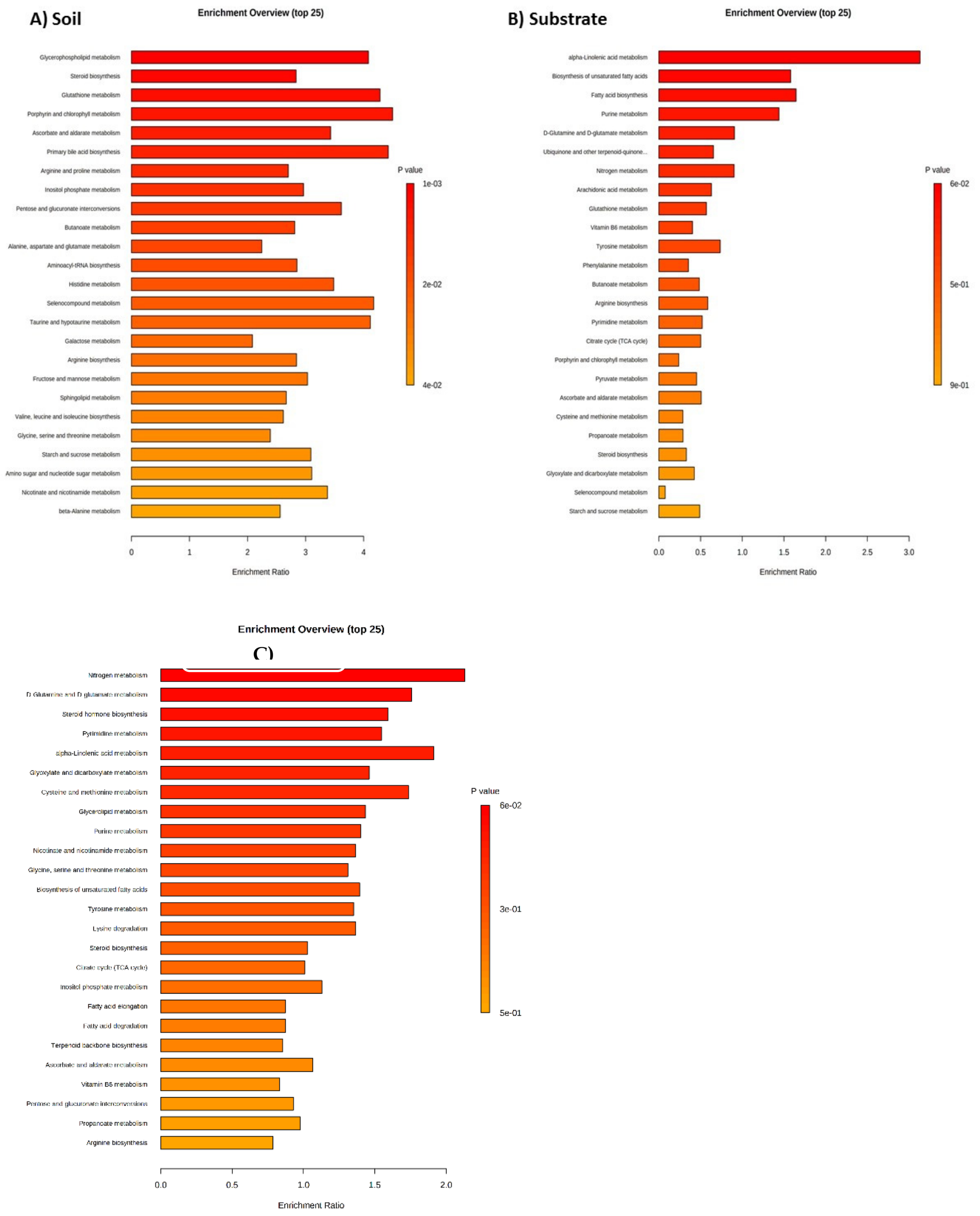

Figure S2. Enrichment overview of top 25 of different metabolites under soil, substrate and hydroponic cultivation system.

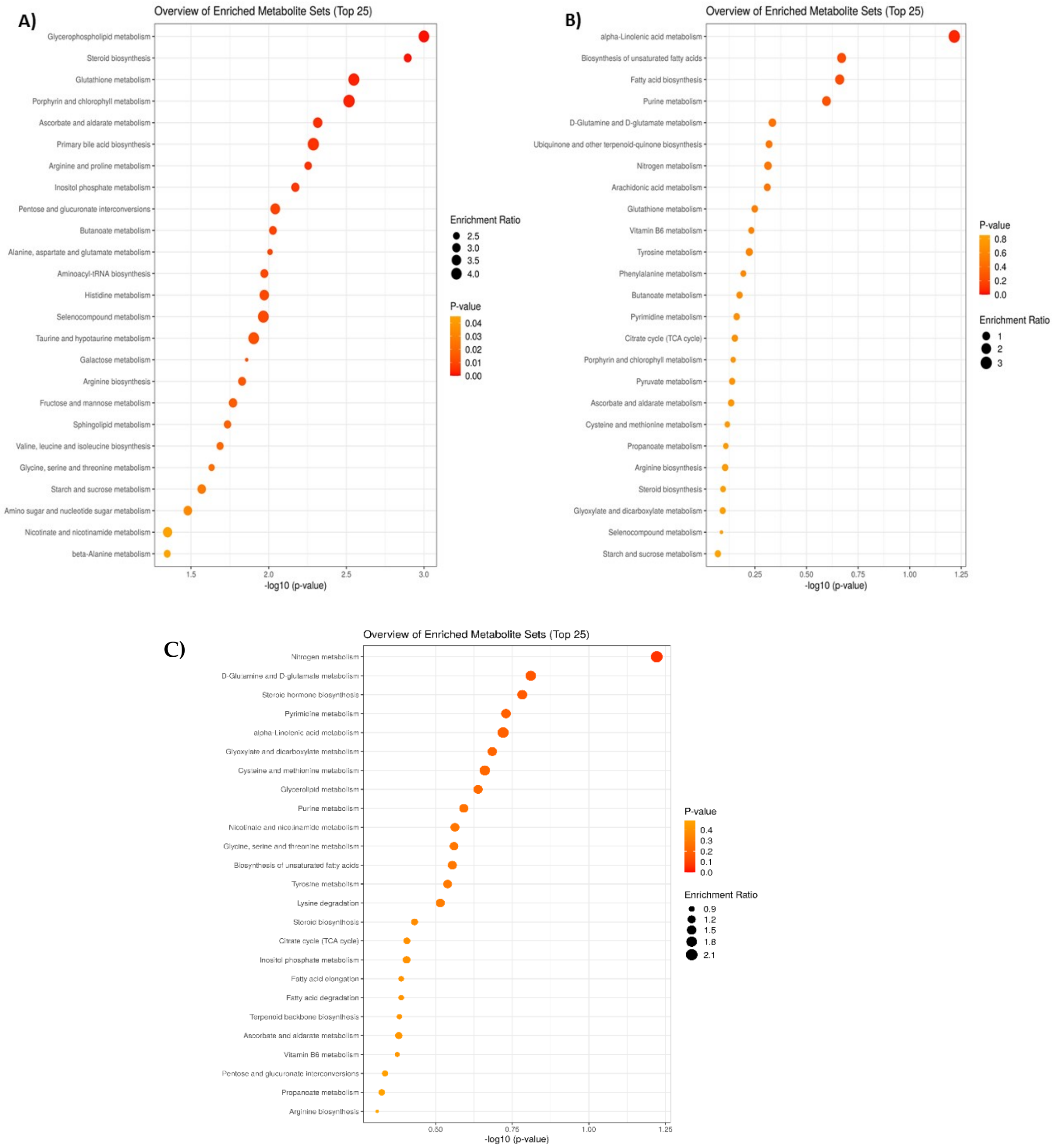

Figure S3. Top 25 of different metabolites sets under soil, substrate and hydroponic cultivation sys-tem.

Note- Different enriched metabolites of lettuce in **Figure A)** represents soil cultivation system , **Figure B)** represents substrate cultivation system and **Figure C)** represents hydroponic cultivation system

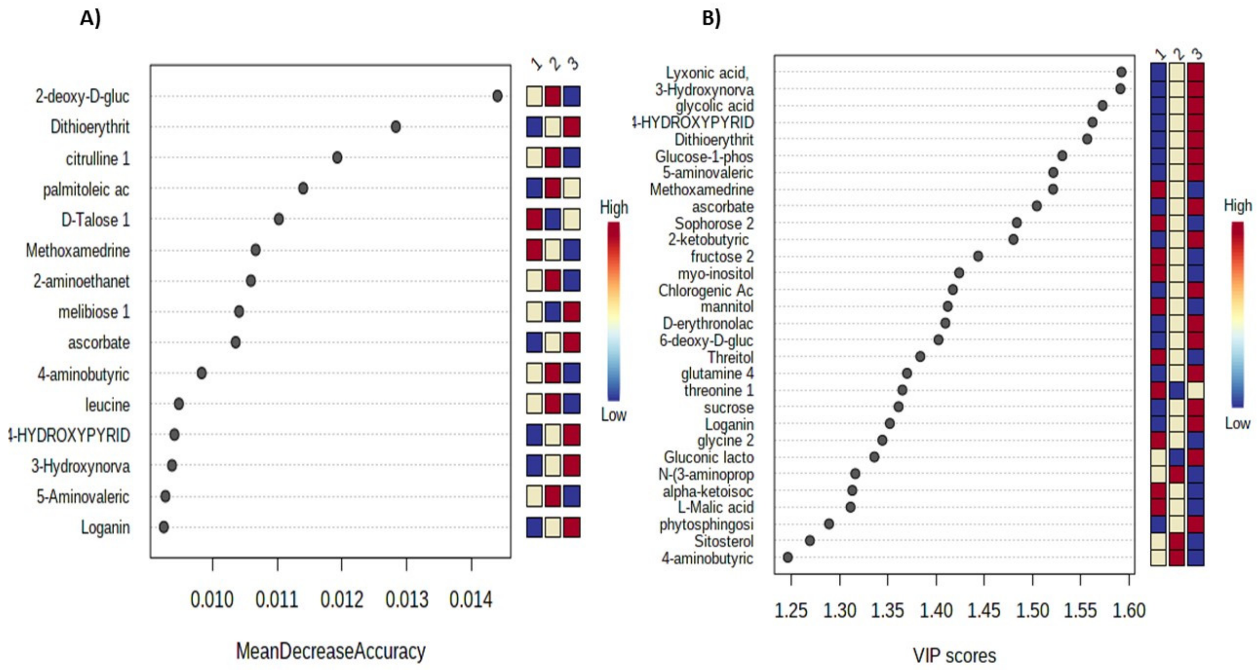

Figure S4. Mean decrease accuracy and VIP score of different metabolites under soil, substrate and hydroponic cultivation system.

Note- Mean decrease accuracy and VIP score of different metabolites of lettuce in **Figure A)** and **B)** number 1, 2 and 3 represent soil, substrate and hydroponic cultivation systems, respectively.

Filename:	metabolites-1654483-supplementary.docx
Directory:	E:\5.16\digital-1703381
Template:	C:\Users\Claudia Timofte\Desktop\Word templates\metabolites-template.dot
Title:	Type of the Paper (Article
Subject:	
Author:	Claudia Timofte
Keywords:	
Comments:	
Creation Date:	5/3/2022 4:12:00 PM
Change Number:	2
Last Saved On:	5/3/2022 4:16:00 PM
Last Saved By:	MDPI 86
Total Editing Time:	5 Minutes
Last Printed On:	5/17/2022 8:58:00 AM
As of Last Complete Printing	
Number of Pages:	5
Number of Words:	774 (approx.)
Number of Characters:	4,516 (approx.)
